# Imaging of Protein Assemblies up to 231 kDa in Tissues with Nano-DESI Mass Spectrometry

**DOI:** 10.1021/acs.analchem.5c05767

**Published:** 2025-12-17

**Authors:** Oliver J. Hale, Helen J. Cooper

**Affiliations:** School of Biosciences, 1724 University of Birmingham, Edgbaston, Birmingham B15 2TT, U.K.

## Abstract

Understanding the distribution of proteins and their assemblies in tissues is a major challenge in spatial biology. Mass spectrometry imaging (MSI) with nanospray-desorption electrospray ionization (nano-DESI) has previously enabled detection, imaging, and identification of intact protein complexes directly from tissues, including protein assemblies and pathological protein–metal complexes in neurodegenerative disease. To date, nano-DESI MSI has been most effective for lower molecular weight (MW) complexes (<100 kDa), with an upper limit of 113 kDa. Here, we demonstrate nano-DESI at molecular weights up to 231 kDa, more than doubling the previous limit, by combining nano-DESI with a new mass spectrometer system architecture designed for higher MW analysis. Both mouse brain and rat kidney tissues were analyzed. Importantly, protein identification by native top-down MS was performed exclusively by use of nano-DESI. That is, complementary techniques for protein identification, such as liquid extraction surface analysis, were not necessary. Both homo- and heteromeric proteoform assemblies were identified in complex with endogenous small-molecule and metal ion cofactors. The developments lead the way to the analysis of larger oligomeric protein assemblies and protein complexes, cementing nano-DESI as a tool for structural biology, and with implications for molecular pathology and drug discovery.

## Graphical abstract

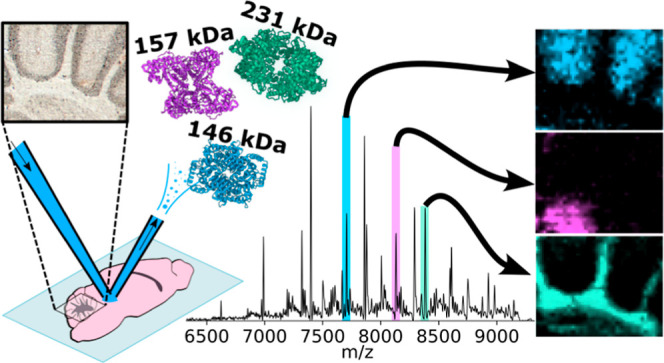

## Introduction

Spatial biology is a rapidly evolving field enabling the understanding of molecular mechanisms that drive physiological processes in tissues.^1,2^ Mass spectrometry imaging (MSI) is a spatial omics technique that can map a wide range of biomolecules, such as metabolites, lipids, and intact proteins. Uniquely, MSI can precisely map chemically distinct forms of individual protein species (proteoforms). We have previously shown that nanospray-desorption electrospray ionization (nano-DESI)^3^ coupled to MS facilitates MSI of intact, noncovalent protein assemblies and complexes directly from the tissue, providing insight into biological function such as their role in neurodegenerative pathology.^4^

A particular challenge for protein MSI is high molecular weight (MW) analysis. The most established MSI methodology, matrix-assisted laser desorption/ionization MSI,^5^ can image denatured proteoforms, but despite a ∼30 year heritage, it remains largely limited to imaging of lower (<30 kDa) MW species. Nano-DESI under denaturing conditions has been used to image proteoforms up to ∼72 kDa^6^ when coupled to charge-detection-MS. Nano-DESI under native-like conditions has achieved imaging of protein complexes with MW ∼100 kDa (94 kDa^7^ and 113 kDa^8^) with conventional MS. Native nano-DESI MSI is generally hindered by poorer signal intensity and signal-to-noise ratio at higher MW. Mass measurement accuracy may also be poor owing to the presence of broad peaks, attributed to incomplete declustering, even when using a high-performance mass analyzer.^9^

Further challenges exist for the in situ identification of protein assemblies and complexes. To avoid inferring proteoform identity from MW alone, separate offline native top-down mass spectrometry (nTDMS) experiments are required. While nano-DESI MS has been used in some cases,^6,10–12^ nTDMS of high MW proteins benefits from offline liquid extraction surface analysis (LESA) MS.^7^ A combination of sample complexity, low signal intensity, and instrument hardware optimized for lower *m*/*z* analysis has hindered nano-DESI for nTDMS at high *m*/*z*. For example, while we have previously reported direct detection of LDHA tetramer from rat liver, poor signal quality precluded imaging the distribution of the assembly.^7^

In this work, we demonstrate nano-DESI MSI for protein assemblies with MW of 125 up to 231 kDa and identify these complexes by nTDMS with the same nano-DESI ion source by harnessing the features of an Orbitrap Ascend Structural Biology mass spectrometer. This mass spectrometer offers high sensitivity *m*/*z* detection up to *m*/*z* 16000, narrow quadrupole isolation (width = 5 *m*/*z*) of precursor ions up to *m*/*z* 8000, and multistage ion manipulation (MS^*n*^). The results presented constitute a more than 2-fold increase in mass range attainable by nano-DESI MSI.

## Methods

### Materials

MS-grade water (catalog no. 10095164) was purchased from Fisher Scientific (Loughborough, UK). HPLC-grade ammonium acetate (catalog no. 15513351) was bought from J.T. Baker (Deventer, Netherlands). The detergent C_8_E_4_ (catalog number T3394) was bought from Merck (Gillingham, UK). Mass spectrometer calibration was performed with FlexMix (catalog number A39239, Thermo Fisher Scientific, San Jose, CA) and ammonium hexafluorophosphate (AHFP, catalogue number 216593, Merck). Nitrogen (>99.995%) and helium (>99.996%) gases used on the mass spectrometer were obtained from BOC (Guildford, UK).

### Animal Tissues

Fresh frozen brains from wild-type mice were the gift of Dr. Richard Mead (University of Sheffield, Sheffield, UK). Sagittal cryosections of 10 μm thickness were prepared from bisected brains by cutting them from the midline with a CM1810 Cryostat (Leica Microsystems, Wetzlar, Germany). Fresh frozen kidney tissue from a vehicle-dosed (0.5% hydroxypropyl methylcellulose (HPMC) and 0.1% Tween 80 in water) adult male Han-Wistar rat was the gift of Dr. Richard Goodwin (AstraZeneca). The animal was euthanized 2 h post dose. Dissection was performed by trained AstraZeneca staff (project license PP77366793, procedure number 3). Sagittal sections of 10 μm thickness were collected, as described above. For both organs, sections were thaw-mounted onto glass microscope slides before storage at –80 °C until analysis. The tissues were thawed under vacuum prior to analysis but were otherwise not further prepared.

### Nano-DESI Mass Spectrometry

All MSI and nTDMS experiments were performed with a home-built nanospray-desorption electrospray ionization (nano-DESI) ion source based on a previously published source design and described previously.^13,14^ A cartoon of nano-DESI is included in Figure S1. Briefly, the nano-DESI probe is composed of two fused silica capillaries. The first delivers a continuously flowing solvent to the sample surface where it forms a dynamic liquid junction bridging the first capillary, the sample, and the second capillary (emitter). Proteins and other endogenous molecules are dissolved from the tissue into this liquid bridge. The emitter aspirates the solvent and dissolved analytes, which are introduced into the mass spectrometer by electrospray ionization. By scanning the tissue sample underneath the nano-DESI probe, we can correlate protein signal abundance with spatial location and map as ion images. The solvent system was 200 mM aqueous ammonium acetate + 0.125% C_8_E_4_ detergent (0.5× critical micelle concentration, CMC), which results in charge-reduced, stabilized noncovalent protein complexes in the gas phase.^15^ Empirical observations in our laboratory suggest that adding C_8_E_4_ to ammonium acetate solutions improves the nano-DESI liquid junction stability and the extraction of protein complexes from the tissue. At 0.5× CMC, soluble and membrane-associated proteins are dissolved, but transmembrane proteins require concentrations exceeding 1× CMC for extraction.^8,16^ The solvent flow rate was typically = 0.6–0.65 μL/min, the probe raster speed was 3–10 μm/s, and the electrospray voltage was tuned to between 800 and 1400 V to achieve electrospray stability with RSD % < 15% (as measured by the tool in Tribrid Tune 4.2) when monitoring the [C_8_E_4_+H]^+^ peak (*m*/*z* 307.2) using the linear ion trap (LIT) mass analyzer.

The nano-DESI ion source was attached to an Orbitrap Ascend Structural Biology Tribrid mass spectrometer (Thermo Scientific, San Jose, CA) configured with the native MS, HMR^*n*+^, ETD, proton transfer charge reduction (PTCR), and UVPD options, referred to herein as “Orbitrap Ascend”. Specifically, the native MS option includes a low-frequency (∼497 kHz versus the standard ∼1090 kHz) RF power supply for the quadrupole mass filter (QMF) for optimized operation at high *m*/*z*. The transmission ion optics operate at ∼1.1 MHz (versus ∼2.4 MHz on the Orbitrap Eclipse mass spectrometer we used previously), and the source prefilter quadrupole is longer to increase collisional cooling of high *m*/*z* ions.^17^ A combination of in-source CID (=200 V) and the source CID compensation factor (=0.07) was set to achieve a broad filtering effect on the ion beam prior to the QMF, Figure S2, Supporting Information.^18^ The Orbitrap Ascend was operated in the “Intact Protein” mode, ion routing multipoles (IRMs) set to the “high pressure” mode (nitrogen bath gas, front-IRM (F-IRM) pressure; 15 mTorr, back-IRM (B-IRM) pressure; 20 mTorr), and LIT helium pressure 2.8 × 10^–5^ Torr. The ion transfer tube was set to 275 °C, and the source pressure was in the range 2.3–2.5 Torr. Note that source pressure is dependent on external factors such as the position of the nano-DESI emitter at the inlet and the ambient temperature, so it varies slightly day to day. Experiment-specific instrument settings are described below, in Tables S1–S3 in the Supporting Information, or with presented data, as appropriate. The Orbitrap Ascend was calibrated using the internal “Auto-ready” ion source to deliver FlexMix and run the automatic calibration procedures for positive ion, negative ion, intact protein, and high mass options. Supplemental calibration with AHFP (for ultrahigh mass calibration only) was performed as required using an external electrospray ionization source.

### Scan Mode Optimization

Three scan modes were evaluated for the best MSI spectral quality (Figure 1) and are discussed in Results and Discussion. Scan mode optimization was automated using an instrument method consisting of the three scan types as interleaved experiments over the course of 15 min. The scan type was switched every ∼3 s (∼12 μm line scan distance). Approx 400 μm × 200 μm of tissue was sampled per scan type. For each scan mode, 27 scans, each comprised of 4 averaged microscans, were collected. Mass spectra presented are the average of the 27 profile mass spectra and are displayed on the same intensity scale. The full performance evaluation method is detailed in Table S1 and Supporting Information. Optimal imaging activation voltage was found by ramping the F-IRM HCD voltage from 0 to 150 V, in 10 V steps (see Figure 1d, Table S2, and Supporting Information). Similarly, this method was automated using an instrument method consisting of the Q-HCD scan type with stepped increments to the HCD voltage defined in the Method Editor’s MS^*n*^ table.

**Figure 1:**
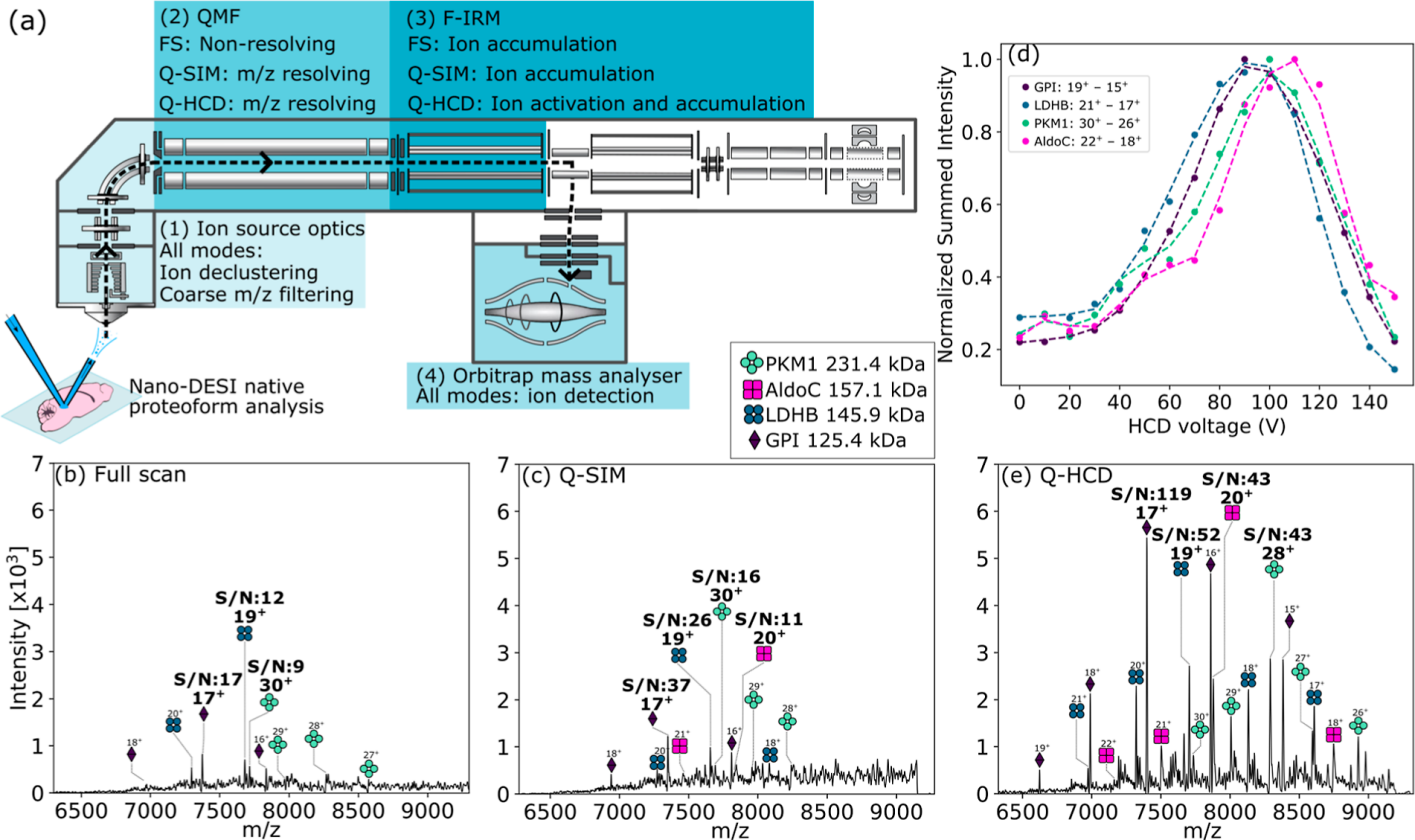
Evaluation of scan modes for nano-DESI analysis of protein complexes at high MW and *m*/*z*. (a) Diagram of the Orbitrap Ascend Structural Biology mass spectrometer and description of device function in each of the three experiment modes. The source ion optics (1) were operated with identical settings for evaluation of all modes. (2) The QMF was operated in the nonresolving mode (Full scan, FS, or resolving mode (Q-SIM, Q-HCD). (3) The F-IRM was used to collisionally decluster ions in Q-HCD mode only. (4) The orbitrap mass analyzer was used for *m*/*z* measurement in all modes. (b) FS nano-DESI spectrum *m*/*z* 6300–9300 and (c) Nano-DESI Q-SIM mass spectrum *m*/*z* 7800 ± 1500. (d) HCD voltage optimization for the Q-HCD scan mode evaluated using the signal intensity of protein complexes in the mouse cerebellum. Signal intensities for multiple charge states per protein complex (indicated in the legend) were summed and normalized. Trendlines: Savitzky–Golay filter (window = 5, polynomial = 3). (e) Nano-DESI Q-HCD mass spectrum *m*/*z* 7800 ± 1500 with 100 V supplemental collision voltage in the F-IRM. Each mass spectrum is displayed on the same intensity scale and are the average of 27 profile mass spectra, each comprised of 4 averaged microscans, representative of a 400 × 200 μm area of tissue per scan type. The scan type was switched every ∼3 s (∼12 μm line scan distance) to avoid spectral differences owing to protein spatial distribution.

### Nano-DESI Mass Spectrometry Imaging

Following optimization of the MS scan mode (see Results), nano-DESI MSI of mouse cerebellum and rat kidney were performed. Instrument parameters are detailed in Table S3, Supporting Information.

### Nano-DESI nTDMS: Ion–Ion Reactions and Collisional Activation

The nano-DESI probe was positioned on serial tissue sections in regions of interest determined from the MSI experiments. The probe was scanned at 1 μm/s within the regions to replenish the protein signal through the sampling of new tissue, which otherwise depletes over ∼ 2 min with a parked probe.

PTCR experiments used perfluoroperhydrophenanthrene (PFPP) as the reagent anion. PTCR reaction times are specified with the relevant nano-DESI-PTCR MS^2^ result but were typically in the range 1–40 ms depending on precursor cation charge and MW. Anion reagent automatic gain control (AGC) target was 2 × 10^5^ charges except in the case of pyruvate kinase M1 (PKM1) where 7 × 10^5^ charges were used. Charge state was fixed to *z* = 3 for all PTCR experiments. Precursor cations were isolated using the QMF (typical width = 5–10 *m*/*z*) for PTCR MS^2^ and the LIT for PTCR MS^3^ (typical width = 50–80 *m*/*z*). The orbitrap analyzer was used for detection with a resolution setting = 7500 (fwhm at *m*/*z* 200).

Beam-type collisional activation (HCD) was performed in the F-IRM with a nitrogen pressure of 15 mTorr (MS^2^) or the B-IRM with a nitrogen pressure of 20 mTorr (MS^3^). Ion isolation was performed using the QMF for HCD MS^2^ (typical width = 5–10 *m*/*z*) and the LIT for HCD MS^3^ (isolation width up to 50 *m*/*z*). Orbitrap resolution was set to 7500 (fwhm at *m*/*z* 200) for protein assembly subunit detection and 240,000 (fwhm at *m*/*z* 200) for mixed subunit/sequence ion detection. HCD voltage used for fragmentation is described with each nano-DESI-HCD MS^*n*^ result but was typically in the range 110–160 V.

### Data Processing

Raw mass spectra were viewed and analyzed in FreeStyle (v1.8.63.0 SP2, Thermo Fisher Scientific). Peak S/N was determined with the in-built tool. For scan mode optimization, Thermo .raw files were first converted to .csv format using the tool in FreeStyle. Then, the intensity data for specified charge states of protein complexes were extracted from the .csv files and plotted with a Matplotlib-based Python script, available at https://github.com/coopergroup-massspec/Q-HCD_voltage_plots.

Brain ion images were processed using the MetaUniDec workflow for protein MSI.^19^ In short, ion images in the imzML format were produced with FireFly (v3.2.0.23, Prosolia Inc.) and passed through imzML Converter^20^ to fix the file for pyimzML compatibility. The fixed imzML file was imported to MetaUniDec^21^ with the imzML import tool (v7.0.0b), and deconvolution was performed on the mass spectrum for each pixel to transform the data from *m*/*z* to mass domain. Deconvolution settings are specified in Table S4. The mass domain data was exported to a new imzML file and viewed with MSiReader (v1.02).^22^ Mass images were generated with a tolerance of ±50 on the mass reported in Table S5 to allow for variability in per-pixel deconvolution. For generating mass images from multiple proteoform charge states in the kidney, a previously described manual processing workflow for Thermo .raw files was used.^7^ Ion images were generated for the *m*/*z* at peak apex ±1 *m*/*z*. This method was used owing to lower S/N in the kidney mass spectra compared to the brain (Figure S3), which was detrimental to UniDec deconvolution. All ion images are shown with 1 order of linear interpolation.

Intact MWs were determined by deconvolution of nano-DESI MSI spectra and nano-DESI-PTCR MS^2^ spectra using UniDec. For identification of proteoforms, a combination of Prosight PC, Prosight Lite, and MASH Native was used.^23,24^ Fragment ions were assigned within a tolerance of 20 ppm from the calculated MW. Proteoforms identified from the mouse brain and rat kidney are described in Tables S5 and S6 and Supporting Information, respectively. Putative assignments were validated manually.

## Results and Discussion

### Improved Spectral Quality with Wide *m*/*z* Isolation and Collisional Activation

Each pixel in a native nano-DESI MS image is a siloed native MS experiment. The quality of each mass spectrum must be balanced with the acquisition time: S/N ratio directly relates to image contrast and is therefore important for interpretation of spatial distributions. On the other hand, data for each pixel must be acquired in a few seconds to keep the total acquisition time practical, e.g., ∼7 h, for a sagittal mouse brain section with 200 μm × 200 μm pixel size. Previously, we have used in-source CID on the Orbitrap Eclipse platform to increase spectral quality in MS imaging of protein complexes.^12^ The effectiveness of this method was diminished for higher MW protein complexes analyzed by nano-DESI (>100 kDa).^7^ We also found wide window (e.g., isolation width = 2000 *m*/*z*) selected ion monitoring (SIM) improved spectral quality but was limited to using LIT isolation on the Orbitrap Eclipse for isolation above *m*/*z* 2000.^4^ The advantage of quadrupole SIM over LIT SIM is that only ions in the defined range are accumulated in the F-IRM and transmitted to the orbitrap, whereas with LIT SIM, all ions are accumulated in the F-IRM before *m*/*z* isolation, resulting in space-charge effects that can lower spectral quality. The system architecture of the Orbitrap Ascend mass spectrometer (Figure 1a) includes a QMF modified for isolation up to *m*/*z* 8000 and a new high-pressure IRM (F-IRM) after the QMF and before the C-trap.

We evaluated three scan modes possible on this system architecture for resulting spectral quality in the context of MSI. First, the full scan mode (denoted Full Scan, Figure 1b) operated with the QMF in the RF-only mode. Second, the SIM mode with quadrupole isolation (denoted Q-SIM, Figure 1c) operated with the QMF set to transmit ions over a selected and wide *m*/*z* range (e.g., *m*/*z* 6300–9300). Note that although the QMF isolation limit in the mass spectrometer software is *m*/*z* 8000, we found it possible to transmit ions up to ∼*m*/*z* 9500 in modes where the QMF was isolating a wide *m*/*z* window. The third mode used collisional activation (HCD) of ions over a quadrupole-selected *m*/*z* range (denoted Q-HCD), which operates with the same QMF-isolated *m*/*z* range as for SIM but with ions being collisionally activated as they transfer from the QMF to the F-IRM. The voltage for Q-HCD was optimized for protein complexes with MW from 125 kDa to 231 kDa in the mouse cerebellum (Figure 1d). A mass spectrum at optimal collision voltage (100 V) is shown in Figure 1e. A similar analysis was performed on protein complexes in rat kidney tissue with HCD = 70 V chosen for MSI (Figure S3, Table S2, Supporting Information).

Native nano-DESI MS spectra from mouse cerebellum tissue, acquired in the three different experiment modes (Full Scan, Q-SIM, and Q-HCD, Figure 1b,c and e respectively), were compared for the range *m*/*z* 6300–9300. For all modes, protein assemblies were detected, and the following were used for performance evaluation: pyruvate kinase (PKM1, 231.4 kDa, homotetramer), Fructose-bisphosphate aldolase C (AldoC, 157.1 kDa, homotetramer), l-lactate dehydrogenase B ((LDHB), 145.9 kDa, homotetramer), and glucose-6-phosphate isomerase (GPI, 125.4 kDa, homodimer); see Table S5, Supporting Information. None of these protein assemblies have previously been identified in native nano-DESI experiments and are discussed further below. The S/N for the most abundant charge state of each of the four protein complexes is labeled in each spectrum.

Absolute signal intensity and S/N was improved in the order Full Scan → Q-SIM → Q-HCD. Q-SIM is generally more sensitive than Full Scan because only ions in the quadrupole-selected *m*/*z* range (*m*/*z* 6300–9300) contribute to the AGC target, which determines the number of ions (strictly, charges) that are trapped and analyzed in the orbitrap; we found modest S/N improvement (∼2 fold) with Q-SIM here. A more dramatic improvement with Q-SIM might have been expected, but its effect is likely diminished by the source ion optics settings employed here. That is, the use of in-source CID and compensation factor scaling (the “voltage rollercoaster”^18^) broadly tunes transmission toward higher *m*/*z* ions, see Figure S2. The Q-SIM mode acts as a secondary filter, rejecting any remaining ions outside the selected transmission range. A Q-SIM method with narrower isolation would likely benefit more since the quadrupole would select a subset of ions transmitted from the ion source optics. Building on the selectivity of Q-SIM, Q-HCD resulted in further signal quality improvement, which we propose to be due to additional ion declustering in the F-IRM. The advantage of additional collisional activation here is that it can be tuned independently of the source ion optics settings. The greatest absolute signal intensity and S/N gain were achieved with ∼100 V HCD voltage (∼2–4 fold improvement versus Q-SIM). Subsequent imaging experiments were performed using the Q-HCD method.

### Nano-DESI MSI of High Molecular Weight Protein Complexes

#### Mouse Brain Cerebellum

Native nano-DESI MS images of the cerebellum of the mouse brain are shown in Figure 2. Four example protein assemblies, which exhibited unique spatial distributions within the structures of the cerebellum, were characterized by nano-DESI nTDMS (see below). That is, it was not necessary to use LESA MS for identification. Homotetrameric PKM1 (Figure 2c, measured MW 231.414 kDa and calculated MW 231.418 kDa) was abundant in the white matter of the arbor vitae. PKM1 is more than double the MW of the previous highest MW protein complex to have been imaged with nano-DESI (aquaporin-0, 113 kDa).^8^ Homotetrameric AldoC (Figure 2d, measured MW 157.058 kDa, calculated MW 157.056 kDa) was abundant only in a region of the cerebellar gray matter, likely a deep cerebellar nucleus. Both PKM1 and AldoC are enzymes in the glycolysis pathway and are essential for supporting the high energy demands of cerebellum function.^25,26^ Their differential expression indicates that specific enzymatic processes on the pathway are dominant in these regions. Two complexes of lactate dehydrogenase (LDH) were also detected, which are also critical to energy production.^25^ A heterotetrameric LDH complex comprising three LDHB subunits and one subunit of glutathionylated LDHA (LDHA_1_B_3_) was highly abundant in the molecular layer (Figure 2e, measured MW 146.212 kDa and calculated MW 146.165 kDa). Finally, homotetrameric LDHB (LDHB_4_) showed the least specific localization of these examples, being broadly distributed throughout the cerebellum with greatest abundance in the white matter (Figure 2f, measured MW 145.926 kDa, calculated MW 145.934 kDa). The two LDH tetramers exhibited very different spatial distributions, suggesting different functions for each modulated by the presence of the LDHA subunit. The subunit composition of LDH tetramers is known to influence enzymatic activity, particularly to bias toward glycolytic or oxidative metabolic pathways and for LDHA and LDHB to be differentially expressed in different brain cell types according to their metabolic requirements.^27^ Notably, LDHB favors conversion of lactate to pyruvate, suggesting this is a major process under aerobic conditions in the arbor vitae.^28^

**Figure 2:**
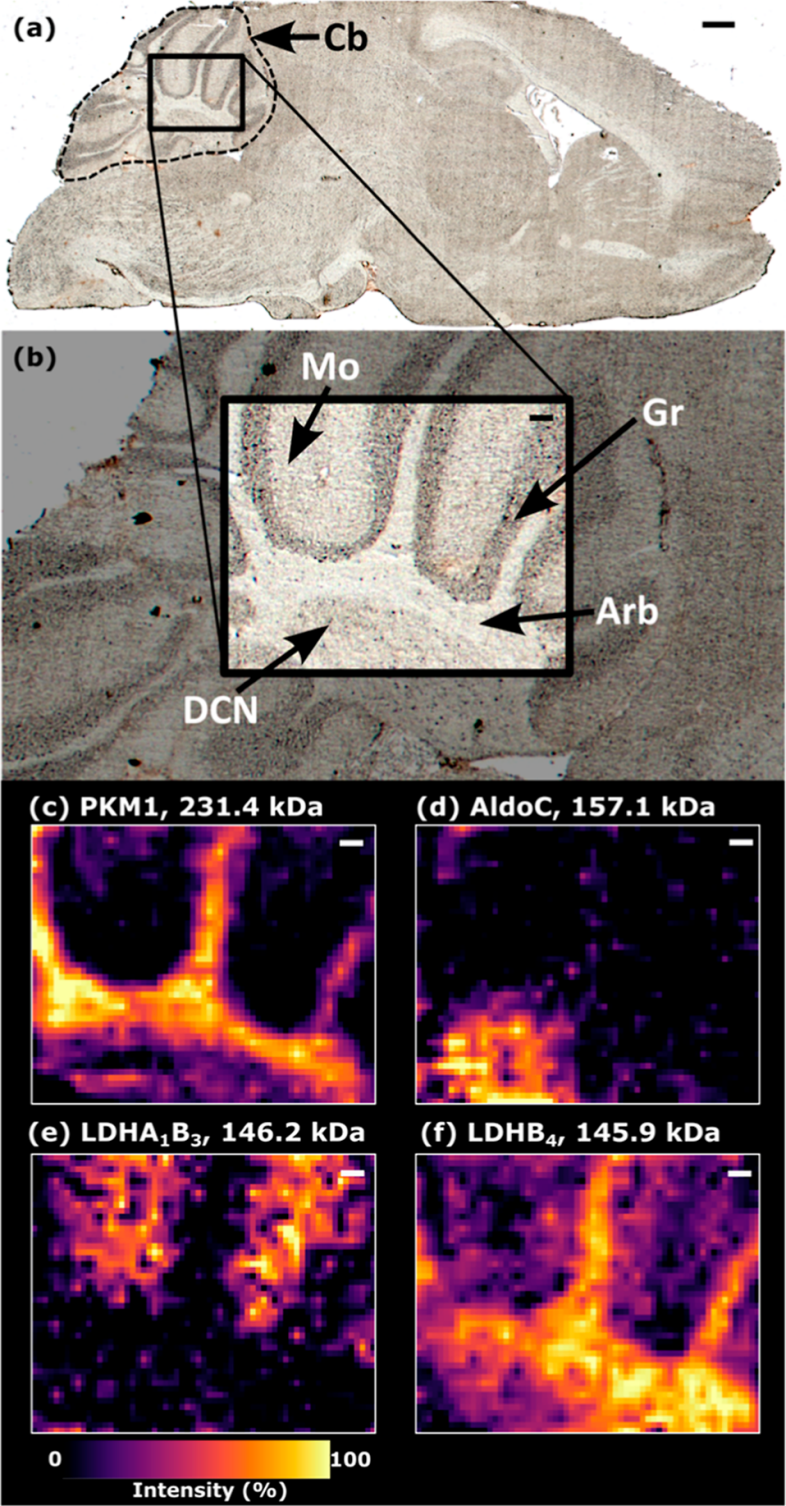
MSI of mouse cerebellum. (a) Brightfield optical image of the mouse brain section in the sagittal plane. (b) Annotated zoom of the cerebellum showing the region imaged by nano-DESI MSI. Nano-DESI MS images for protein complexes in the cerebellum; (c) PKM1, 231.4 kDa, homotetramer (charge states 30^+^-26^+^); (d) aldolase C, 157.1 kDa, homotetramer (charge states 21^+^-19^+^); (e) LDHA_1_B_3_ heterotetramer, 146.2 kDa (charge states 21^+^-19^+^); and (f) LDHB, 145.9 kDa (charge states 21^+^-18^+^), homotetramer. Annotations: Mo: molecular layer, Gr: granular layer, Arb: arbor vitae white matter, and DCN: deep cerebellar nuclei. MSI pixel size: 46 × 50 μm. Scale bar (a) and 500 μm, (b–f) 100 μm. Mass image tolerance = deconvoluted MW ± 25 Da.

#### Rat Kidney

Nano-DESI MSI of higher MW proteins in kidneys is shown in Figure 3. As above, protein assemblies were identified by nano-DESI nTDMS (see below). The imaged region covered cortex, outer medulla, and inner medulla tissue and featured vasculature (Figure 3a,b). The LDHA_4_ complex (Figure 3c, measured MW 145.430 kDa, calculated MW 145.447 kDa) showed a localized distribution indicating abundance in specific structures of the nephron in the inner and outer medulla. The abundance of homotetrameric LDHA indicates anaerobic pyruvate to lactate conversion in these specific regions. Complexes containing LDHB, favoring lactate to pyruvate conversion, are expressed throughout other tissue regions. The heterocomplex LDHA_1_B_3_ (Figure 3d, measured MW 145.935 kDa, calculated MW 145.932 kDa) was abundant in the cortex and inner medulla but not detected in the outer medulla. The LDHB_4_ homotetramer complex (Figure 3e, measured MW 146.092 kDa, calculated MW 146.094 kDa) was most abundant throughout the inner medulla. Previous imaging of LDHA and LDHB using antibodies lacked specificity to complex subunit composition,^29^ whereas imaging by MW here provides that specificity. Transketolase (Tkt, Figure 3f, measured MW of 136.278 kDa, calculated MW of 136.264 kDa) in complex with its cofactors Mg^2+^ and thiamine pyrophosphate (MW ∼422 Da, a derivative of vitamin B_1_) exhibited abundance in the inner medulla and cortex. Tkt is an essential enzyme in the pentose phosphate metabolic pathway.^30^ Homotetrameric cystathionine gamma-lyase (CGL) in complex with its cofactor, pyridoxal phosphate (PLP), the active form of vitamin B_6_ (Figure 3g, measured MW 175.584 kDa; calculated MW 175.400 kDa), was abundant in the outer medulla. CGL is an important enzyme in the *trans*-sulfuration pathway for biosynthesis of cysteine and glutathione and thus serves to protect the kidney from oxidative stress.^31^ The outer medulla is more susceptible to hypoxia, and thus oxidative stress, relative to other regions of the kidney.^32^ The last example image is attributed to a 185 kDa protein localized to vasculature (Figure 3h, measured MW 185.202 kDa); this protein remains unidentified despite a rich fragment ion spectrum, suggesting additional molecular complexity that we are unable to solve with existing data analysis tools, see the discussion below.

**Figure 3:**
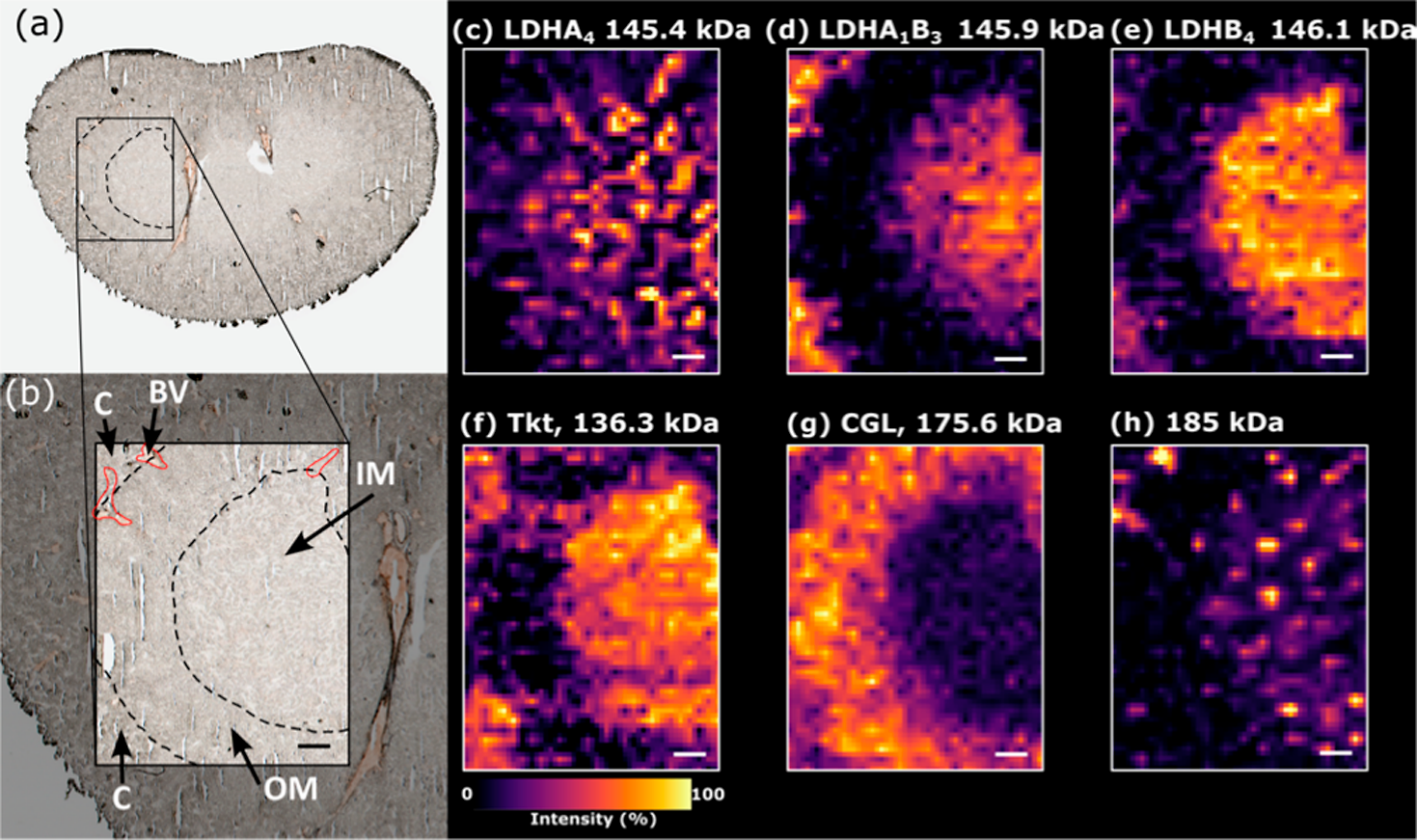
MSI of rat kidney. (a) Brightfield optical image of the rat kidney section in the sagittal plane. (b) Annotated zoom of the kidney showing the region imaged by nano-DESI MSI. Nano-DESI MS images for (c) LDHA_4_ (charge states), (d) LDHBA_1_B_3_ (charge states 21^+^-18^+^), (e) LDHB_4_ (charge states 21^+^-17^+^), (f) transketolase in complex with Mg^2+^ and thiamine pyrophosphate (charge states 19^+^ & 18^+^), (g) CGL in complex with pyridoxal phosphate (charge states 25^+^-22^+^), and (h) an unidentified 185 kDa protein (charge states 24^+^-21^+^). Annotations: BV: blood vessel (larger BVs in red borders), C: cortex, OM: outer medulla, and IM: inner medulla. MSI pixel size: 144 × 150 μm. Scale bar (b–h) 500 μm. Ion image *m*/*z* tolerance = most intense data point for each charge state within a *m*/*z* ± 1 window.

### Identification of Brain and Kidney Complexes by Nano-DESI nTDMS

Each protein complex in the mouse brain cerebellum and rat kidney was characterized by nano-DESI nTDMS (Figures 4 and 5, respectively). PTCR MS^2^ was used to validate the intact molecular weights calculated from MSI data (Table S5 and Table S6, Supporting Information). HCD MS^2^ was used to obtain sequence fragments for proteoform identification.

**Figure 4:**
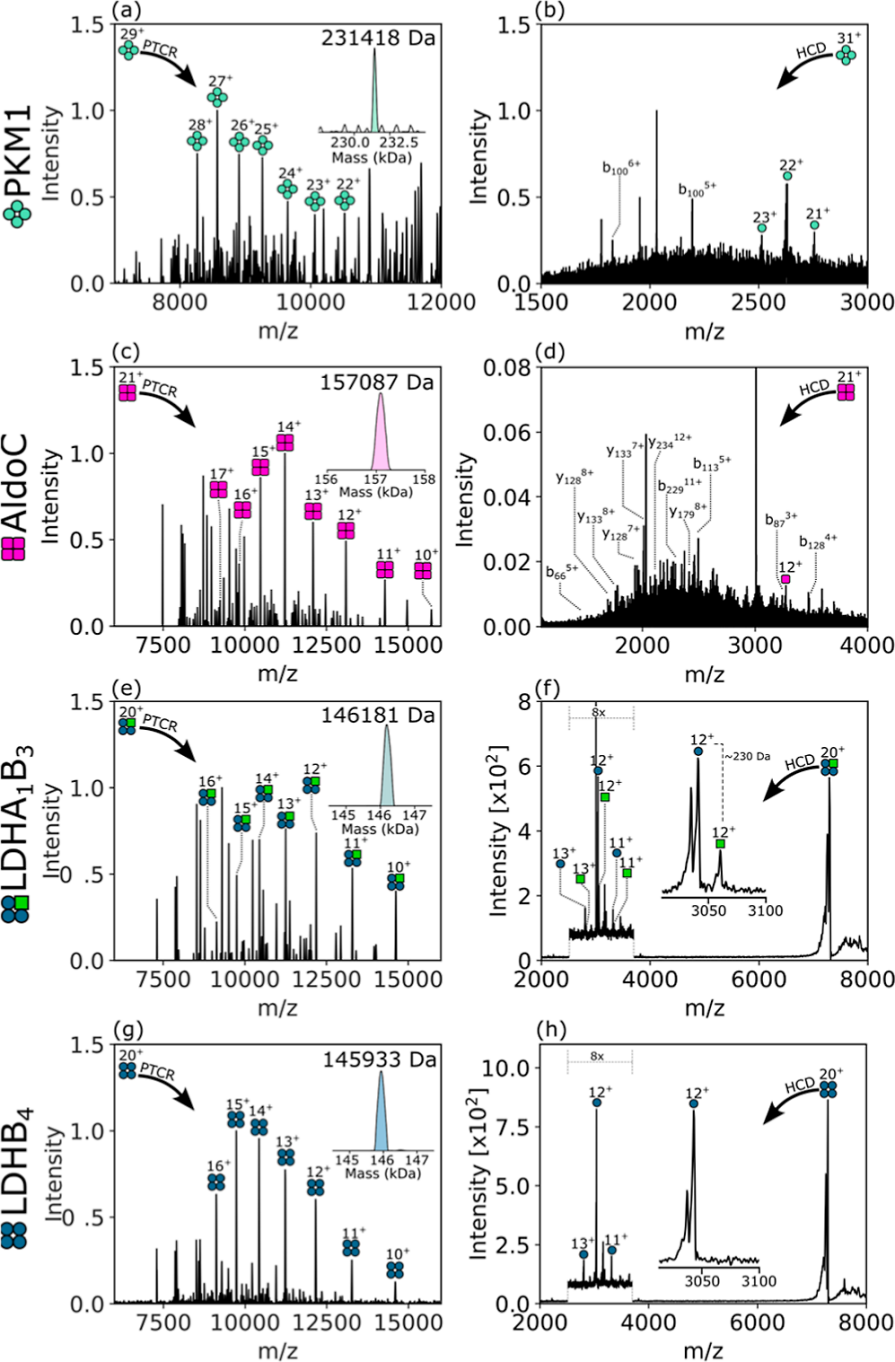
Nano-DESI MS^2^ spectra of protein complexes from brain images. (a) PTCR MS^2^ of PKM1 (*m*/*z* 7982^29+^ ± 10, 2 ms), (b) HCD MS^2^ of PKM1 (*m*/*z* 7476^31+^ ± 2.5, 138 V), (c) PTCR MS^2^ of AldoC (*m*/*z* 7479.6^21+^ ± 2.5, 35 ms), (d) HCD MS^2^ of AldoC (*m*/*z* 7481^21+^ ± 2.5, 142 V), (e) PTCR MS^2^ LDHA_1_B_3_ (*m*/*z* 7309^20+^ ± 2.5, 35 ms), (f) HCD MS^2^ of LDHA_1_B_3_ (*m*/*z* 7310^20+^ ± 2.5, 135 V), (g) PTCR MS^2^ of LDHB_4_ (*m*/*z* 7297^20+^ ± 5, 40 ms), and (h) HCD MS^2^ of LDHB_4_ (*m*/*z* 7297^20+^ ± 2.5, 135 V). Deconvolution result for each PTCR MS^2^ spectrum shown as an inset. PTCR reaction time (ms) and HCD voltage (V) for each experiment are in brackets.

**Figure 5:**
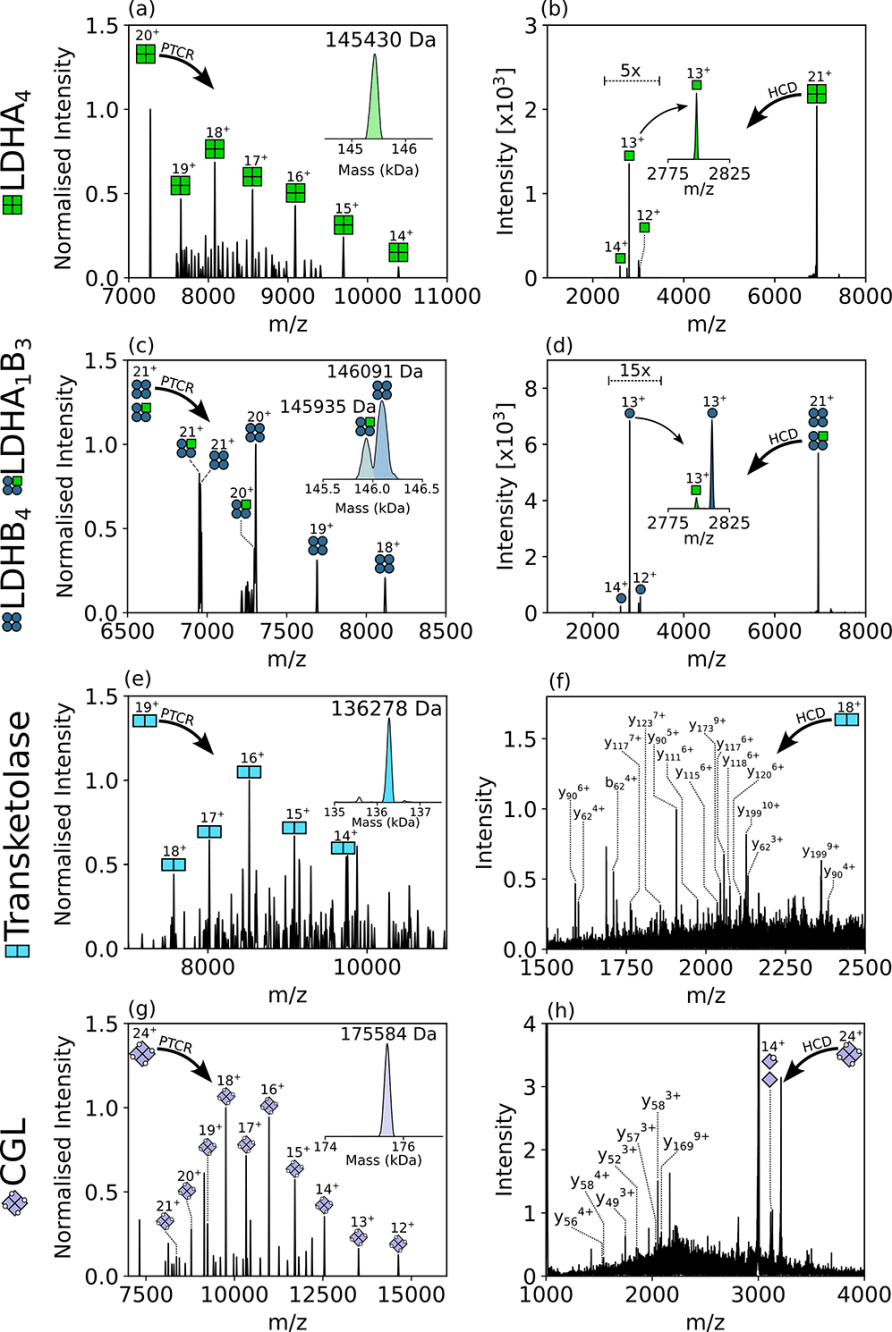
Nano-DESI MS^*n*^ spectra for protein complexes from kidney images. (a) PTCR MS^2^ of LDHA_4_ (*m*/*z* 7272^20+^ ± 5, 1.8 ms), (b) HCD MS^2^ of LDHA_4_ (*m*/*z* 6928^21+^ ± 2.5, 110 V), (c) PTCR MS^2^ of LDHB_4_ and LDHA_1_B_3_ (*m*/*z* 6961 ± 10, 1 ms), (d) HCD MS^2^ of LDHB_4_ (*m*/*z* 6960^21+^ ± 2.5, 110 V), (e) HCD-PTCR MS^3^ of transketolase homodimer complex (*m*/*z* 7500±3000, 70 V → *m*/*z* 7175 ± 30, 2 ms) and (f) HCD MS^2^ of transketolase homodimer complex (*m*/*z* 7572^18+^ ± 5, 135 V). (g) PTCR MS^2^ of CGL homotetramer (7316^24+^ ± 2.5, 3 ms), (h) deconvoluted HCD-PTCR MS3 of CGL homotetramer (*m*/*z* 7636^23+^ ± 10, 125 V → *m*/*z* 3133^14+^ ± 40, 1.2 ms), and (h) HCD MS^2^ of the transketolase homodimer complex (*m*/*z* 7572^18+^ ± 5, 135 V). Deconvolution result for each PTCR spectrum is shown as an inset. PTCR reaction time (ms) and HCD voltage (V) for each experiment are in brackets.

#### Native TDMS of Mouse Brain Complexes

PTCR MS^2^ of PKM1 gave a MW of 231.418 kDa (Figure 4a, calculated MW = 231.418 kDa). HCD MS^2^ of PKM1 (Figures 4b, S4, Table S7) released monomer subunits that were detected in charge states 23+, 22+, and 21+ and deconvoluted to 57852 Da (calculated MW = 57854 Da), confirming the homotetrameric stoichiometry of the precursor complex. A single sequence ion was detected in two charge states (b_100_^6+^ and b_100_^5+^), resulting from cleavage between Asp and Pro (a site known to be favored under native conditions^33^). Despite the lack of sequence coverage by fragment ions, the combined evidence (i.e., tissue specificity (abundance in cerebellum), intact MW, subunit MW, and cleavage at a specific residue pair) leads to confident identification. PTCR MS^2^ of AldoC revealed MW = 157.087 kDa (Figure 4c, calculated MW = 157.056 kDa). The HCD MS^2^ spectrum for AldoC revealed poor subunit ejection but was rich in sequence ions, as reported by others,^34^ confirming its identity (Figures 4d, S5, Table S8). The heteromeric LDHA_1_B_3_ complex was measured to have MW = 146.181 kDa (Figure 4e, calculated MW = 146.165 kDa). In the HCD MS^2^ spectrum of LDHA_1_B_3_, the stoichiometry of the heterotetramer is reflected in the relative intensity ratio of the subunits, approximately 3:1 LDHB:LDHA (Figure 4f). The LDHA subunit was higher in MW than predicted from its sequence (measured MW = 36.714 kDa, calculated MW = 36.410 kDa, Δ = 304 Da) but HCD MS^3^ fragmentation of the subunit at *m*/*z* 2825^13+^ produced LDHA *y*-ions (Figure S6, Table S9). The MW difference of ∼304 Da indicates glutathionylation of one of the cysteine residues in the LDHA subunit. The detected *y*-ions eliminated C292 as a glutathionylation site, leaving C34, C83, C130, C162, and C184 as candidates as no b-ions were detected. We speculate that the role of LDHA glutathionylation may regulate its activity and protect it from the oxidative cellular environment of the cerebellum, as is known for other proteins.^35^ PTCR MS^2^ resulted in a measured MW = 145.933 kDa for LDHB_4_ (Figure 4g, calculated MW = 145.934 kDa). HCD MS^2^ of LDHB_4_ (Figures 4h, S7, Table S10) confirmed LDHB as the component subunit. LDHB fragmented to produce low MW b-ions and a series of higher MW *y*-ions. The GPI homodimer used in the HCD voltage optimization experiment was measured to have MW = 125.357 kDa (calculated MW = 125.357 kDa) with PTCR MS^2^, and its identity was confirmed through HCD MS^2^ (Figure S8, Table S11). Ejected subunits were not detected for GPI.

#### Native TDMS of Kidney Complexes

LDHA_4_ was measured to have a MW of 145.430 kDa by PTCR MS^2^ (calculated MW = 145.447 kDa, Figure 5a), and HCD MS^2^ confirmed its homotetrameric stoichiometry and putative identity by intact subunit mass (Figures 5b, S9). No sequence fragments were observed and could not be obtained by HCD MS^3^, likely due to the low intensity of precursor ions. Likewise, PTCR MS^2^ measured LDHA_1_B_3_ and LDHB_4_ to have molecular weights of 145.935 Da (calculated MW = 145.932 kDa) and 146.092 kDa (calculated MW = 146.094 kDa), respectively (Figure 5c). Note that LDHA_1_B_3_ and LDHB_4_ complexes were isolated for PTCR within the same window owing to their close *m*/*z* values in their 21^+^ charge state. The homotetrameric stoichiometry of LDHB_4_ (Figure 5d) and sequence information were obtained through HCD MS^2^ (Figures 5d, S10, Table S12). Within this spectrum, a signal for the 13^+^ charge state of LDHA was also detected, indicating the presence of the LDHA_1_B_3_ heterotetramer. The measured subunit MWs of LDHA and LDHB combine in a 1:3 ratio to give the MW of the LDHA_1_B_3_ complex. Unlike in the cerebellum, the LDHA subunit was not detected with glutathionylation.

The MW of the transketolase (Tkt) complex was measured as 136.278 kDa (calculated MW = 136.264 kDa), consistent with the homodimeric form of Tkt bound to two magnesium ions (∼24 Da) and two thiamine diphosphate (∼422 Da) cofactors. Tkt is an essential enzyme involved in the pentose phosphate pathway, and divalent metal cations cofactors are required for its function, although there is debate over whether the physiological metal ion is Ca^2+^ or Mg^2+^.^36^ For measurement of Tkt MW, collisional activation was applied to increase precursor ion signal intensity in a manner similar to the approach used for the imaging experiments above (HCD voltage = 70 V, see Figure S3) before performing PTCR MS^3^ (HCD-PTCR MS^3^, Figure 5e). The measured MW is +14 Da versus that calculated with 2*x* Mg^2+^ ions, potentially indicating binding of Mg^2+^ and Ca^2+^ ions in tissue, although this may be further convoluted by nonspecific adduction with Na^+^ and K^+^ ions The HCD MS^2^ spectrum (Figures 5f, S11, Table S13) is populated by Tkt *y*-ions, but intact transketolase monomers were not detected, which prevented further measurement of the metal ion + subunit MW.

The protein complex identified as CGL was measured to have an intact MW of 175.584 kDa (calculated MW = 175.400 kDa) by PTCR MS^2^ (Figure 5g). Putative subunit signals in the HCD MS^2^ spectrum (Figures 5h, 6a) suggested a tetrameric stoichiometry but were not isotopically resolved, and additional charge states were not detected to enable deconvolution. HCD-PTCR MS^3^ confirmed that the subunit signals were in the 14^+^ charge state (Figure 6b) and confirmed the tetrameric stoichiometry. Sequence ions revealed the protein identity as CGL (Figures 5h, S12, and Table S14). The deconvoluted subunit mass spectrum (Figure 6c) revealed CGL subunits bound to a cofactor and in an unbound state, resulting in a mass difference of ∼240 Da between the two major signals. The signal at higher MW (43.850 kDa) is consistent with the CGL subunit bound to the physiological cofactor PLP (MW ∼245 Da). The peak at lower MW (42.620 kDa) is indicative of the gas-phase dissociation of that cofactor during collisional activation at the MS^2^ level, resulting in product ions that were also selected in the MS^3^ isolation window. Notably, the CGL tetramer detected here has an MW discrepancy (+184 Da) to the calculated MW for a tetramer bound to 4*x*PLP molecules. This difference indicates additional molecular constituents were present in the complex that were dissociated from the CGL subunits during HCD MS^2^, preventing further identification. The homotetrameric complex of mitochondrial acetyl-CoA acetyltransferase was identified by nano-DESI-HCD MS^3^ (Figure S13, Table S15) and used in the HCD voltage optimization only.

**Figure 6:**
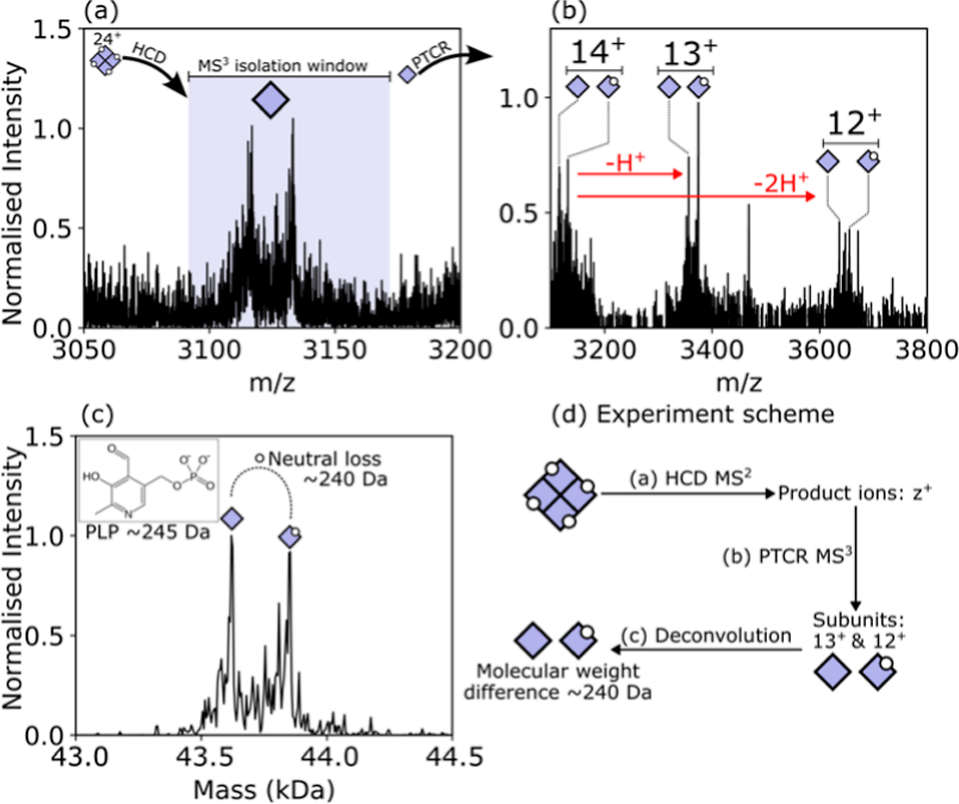
(a) Mass spectrum HCD MS^2^ product ions assigned to CGL subunits. The highlighted region was selected for (b) HCD-PTCR MS^3^, which confirmed the MS^2^ product ion charge state as 14^+^. (c) Deconvolution of the HCD-PTCR MS^3^ spectrum revealed a MW difference of approximately 240 Da between subunit signals, approximately the MW of PLP. (d) Scheme summarizing the experiment workflow.

The 185 kDa protein imaged in Figure 3 remains unidentified, although the MW was confirmed through nano-DESI-PTCR MS^2^ (185.202 kDa, Figure S14). This finding highlights a continuing challenge for nTDMS from biological samples; highly populated product ion spectra (e.g., the HCD spectrum of the 185 kDa protein in Figure S15) may not yield reasonable proteoform identifications. Factors such as chimeric MS^*n*^ spectra and the vast search space to consider for PTMs compound the issue. New approaches that improve the specificity of protein complex MS^*n*^ together with narrow *m*/*z* isolation will need to be developed, with ion mobility spectrometry and gas-phase charge manipulation offering potential for separating proteoforms and avoiding chimeric spectra.^37,38^ Data analysis software also needs advancements. Packages including Prosight Native^39^ and MASH Native^24^ are powerful native top-down sequencing suites but do not use all information available from an on-tissue experiment to narrow down proteoform identification. Recently introduced approaches use fragment first analysis, isotopic pattern recognition, and PTM-aware data analysis.^40,41^ From the imaging perspective, additionally considered information could include correlation of tissue spatial distribution and the proposed proteoform identity to filter false-positive hits, e.g., erroneous “identification” of a proteoform expressed exclusively in the brain when analyzing kidney tissue. Currently, we perform this assessment manually, but it could be automated with reference to the information available in databases such as UniProt.

The transketolase and GPI examples in this work highlight another challenge for confident identification: In some cases, intact subunits are not observed following HCD, precluding definitive confirmation of stoichiometry.^34^ Surface-induced dissociation (SID) may solve this problem as it results in product ions reflective of quaternary structure of complexes more reliably than slow-heating collisional activation such as HCD.^42^ SID could also simplify the analysis of heteromers by ejecting all subunits into a *m*/*z* range suitable for MS^*n*^. The heterotetramer LDHA_1_B_3_ was identified in both mouse cerebellum and rat kidney but containing different proteoforms, unmodified in kidney and glutathionylated in brain. While both subunits of this complex were detected following HCD, in some cases, asymmetric charge partitioning can result in low charge, high *m*/*z* monomer product ions which are unsuitable for further top-down fragmentation. Incorporation of SID into a MS^*n*^-capable mass spectrometer could enable more confident and deep analysis of heterocomplexes.

Often, precursor ion signal intensity is the greatest challenge to a successful on-tissue MS^*n*^ experiment, especially where direct fragmentation of the complex does not readily yield sequence fragments and MS^3^ is required.^34^ MS^*n*^ of high MW protein complexes directly from tissue remains very challenging. Data of sufficient quality for identification requires long experiments (more than 15 min) for even “abundant” precursor ions (i.e., ∼1 × 10^3^–1 × 10^4^ (orbitrap normalized level)) derived from the tissue. It is also difficult and time-consuming to find an appropriate HCD voltage for low-intensity precursor ions since product ion spectra need minutes of data collection to build fragment signals. At least two optimized activation values or stepped activation methods^43^ are desirable for complexes: (i) to eject subunits to determine stoichiometry (if possible) and (ii) for generating sequence ions. Any solutions that make for more predictable ion activation in the native TDMS space would be extremely valuable. Comprehensive sequence coverage will likely require generational technological advancements across all fronts.

Finally, it is worth noting that the MW ceiling reported here is a consequence of the fact that narrow width ion quadrupole isolation on the Orbitrap Ascend is limited to *m*/*z* 8000. While higher *m*/*z* isolation is available on other instruments, they do not currently provide the same versatility (MS^*n*^ and/or ion–ion reactions) or performance (e.g., quadrupole isolation width = 5 *m*/*z*), both of which are invaluable for samples with the complexity of tissue.^44,45^ Technology developments that extend the *m*/*z* isolation range in parallel with MS^*n*^ capabilities will undoubtedly further raise the MW limit for native ambient mass spectrometry.

## Conclusions

We have demonstrated native MS imaging and native TDMS with nano-DESI for protein complexes in mouse and rat tissues spanning the MW range 125–231 kDa. The MW range possible with nano-DESI MSI is now more than double the previous highest MW, strengthening its position as a tool for in situ structural biology. There now exists an overlap between the higher MWs accessible with native nano-DESI MSI and the lower MWs accessible with cryo-electron tomography pointing to future studies that could combine these two technologies.^46^

A key advance reported here is the identification of the protein assemblies solely by use of nano-DESI. Previously, we have relied on LESA MS for protein identification as an adjunct to nano-DESI for visualization of protein distribution. It is also important to note that, while to date the protein assemblies detected and identified by native ambient mass spectrometry have predominantly been homomeric, we identified heteromeric assemblies in this work.

Despite the improvements realized here, requiring collisional activation of protein complexes for improving signal quality is counterintuitive when one of the goals of native MS is to retain noncovalent interactions, which may be disrupted. Although we have detected complexes intact with their endogenous cofactors with native stoichiometries, there will likely be more delicate complexes that will not tolerate activation. We will explore alternative methods for boosting the signal intensity in future work. Nano-DESI MSI has already revealed new information on lower MW (∼30 kDa) protein complexes in a neurodegenerative proteinopathy.^4^ With a much higher MW range now accessible, imaging and identification of pathological protein candidates, such as early stage oligomers, throughout various tissue types will be possible.

## Supplementary Material



## Notes

Additional experimental details (instrument settings and deconvolution software parameters), supporting mass spectra, and details of protein assignments (including sequence coverage and fragment ion assignments) (PDF)
