# Supplementary material for: Imaging of Protein Assemblies up to 231 kDa in Tissues with Nano-DESI Mass Spectrometry
Source: Anal Chem. 2025 Dec 17;97(51):28343–52. doi: 10.1021/acs.analchem.5c05767 (PMC12756848; doi:10.1021/acs.analchem.5c05767)
Supplement: Supplementary file 1 [file ac5c05767_si_001.pdf]

SUPPORTING INFORMATION FOR "IMAGING OF PROTEIN ASSEMBLIES UP TO 231 KDA IN TISSUE WITH NANO-DESI MASS SPECTROMETRY."

Oliver J. Hale and Helen J. Cooper*

School of Biosciences, University of Birmingham, Edgbaston, Birmingham B15 2TT, UK.

*To whom correspondence should be addressed. H.J.Cooper@bham.ac.uk

Table of contents: Supporting Figures

Figure S1: cartoon of the nano-DESI ion source. 2
Figure S2: nano-DESI full scan mass spectrum for the range m/z 1000 – 9500 2
Figure S3: HCD voltage optimisation for protein complexes in rat kidney 4
Figure S4: Sequence ions of PKM1 6
Figure S5: Sequence ions for mouse AldoC 7
Figure S6: MS3 of GSH-LDHA 8
Figure S7: Sequence ions of LDHB 9
Figure S8: nano-DESI MS^2 analysis of mouse GPI 11
Figure S9: Isotopic distribution of LDHA 13+ subunit ejected from $LDHA_4$ 13
Figure S10: Sequence ion coverage of rat LDHB.. 13
Figure S11 Sequence ion coverage for the sequence of rat transketolase. 14
Figure S12: Sequence ion coverage of rat CGL. 16
Figure S13: HCD MS^3 spectrum and sequence ions for Acat1 17
Figure S14: PTCR MS^2 spectrum of m/z 7718^{24+} 18
Figure S15: HCD MS^2 spectrum of m/z 7718^{24+} 19

Table of contents: Supporting Tables

Table S1: Experimental settings for scan mode evaluation. 3
Table S2: Experimental settings for Q-HCD voltage optimisation experiments. 3
Table S3: Instrument method details for nano-DESI MSI. 3
Table S4: MetaUniDec settings for deconvolution 4
Table S5: mouse brain protein complexes identified in this work. 5
Table S6: rat kidney protein complexes identified in this work. 5
Table S7: b_{100} sequence ion of PKM1. 6
Table S8: Detected sequence ions for mouse AldoC. 7
Table S9: detected sequence ions for LDHA 8
Table S10: sequence ions for mouse $LDHB_4$. 10
Table S11: Sequence ions for mouse GPI. 12
Table S12: detected sequence ions for rat LDHB. 14
Table S13: detected sequence ions for rat transketolase. 15
Table S14: detected sequence ions for rat CGL. 16
Table S15: detected sequence ions for rat Acat1. 18

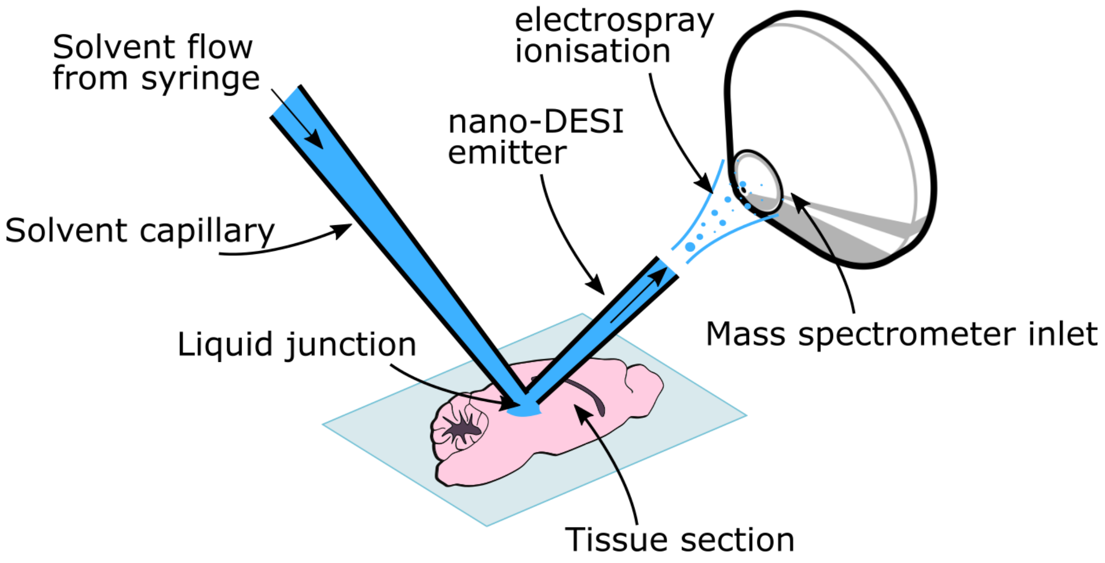

Figure S1: cartoon of the nano-DESI ion source. Note that in practice the emitter exit is placed within a few hundred micrometres of the mass spectrometer inlet for aspiration of solvent using the mass spectrometer vacuum.

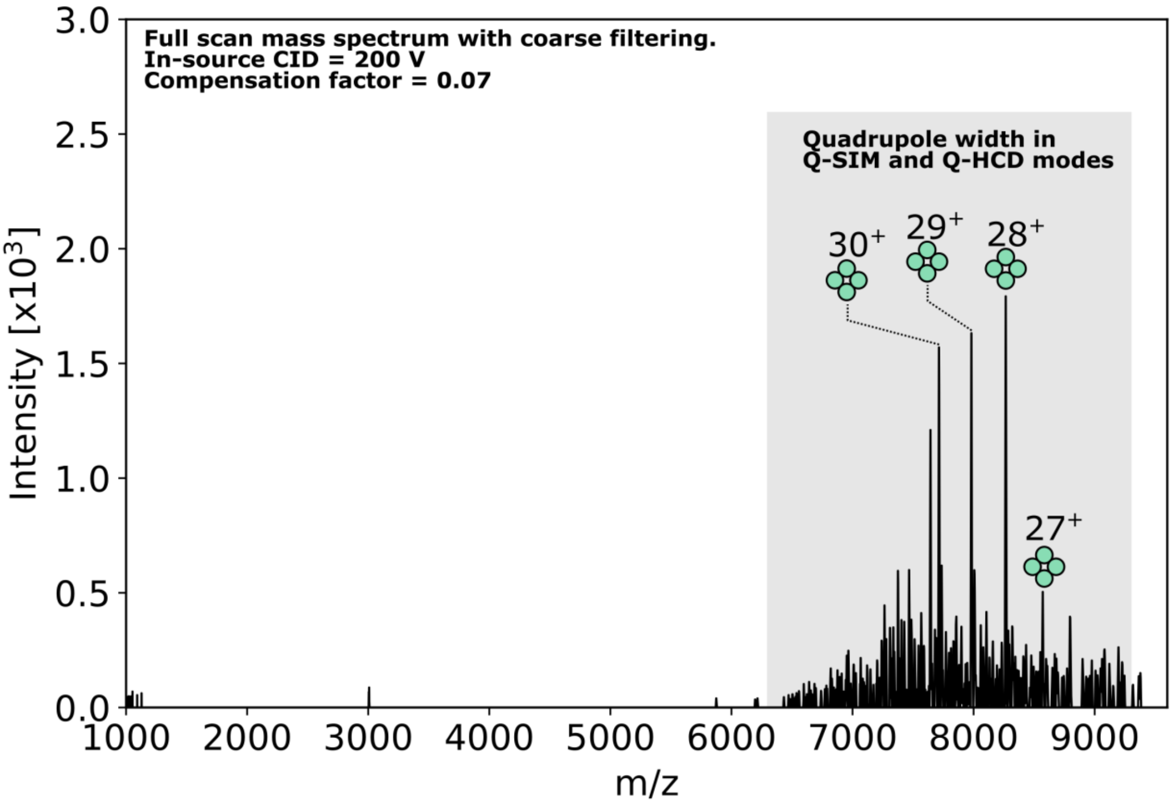

Figure S2: nano-DESI full scan mass spectrum for the range m/z 1000 – 9500 in mouse brain white matter with in-source CID (200 V) and source CID compensation factor (0.07) filtering applied. The effect of these source conditions is to transmit a broad m/z range of ions of approximately m/z 6000 – 9500 while abundant ions at low m/z are rejected. Gray shading indicates the quadrupole isolation window applied in subsequent Q-SIM and Q-HCD experiments. Peaks corresponding to pyruvate kinase tetramer charge states 30+ - 27+ are labelled.

Table S1: Experimental settings for scan mode evaluation.

Setting	Full scan	Q-SIM	Q-HCD
Nano-DESI flow rate (µL/min)	0.65	0.65	0.65
Nano-DESI probe velocity (µm/s)	3	3	3
Nano-DESI spray voltage (V)	1275	1275	1275
In-source CID (V)	200	200	200
Source CID compensation factor	0.07	0.07	0.07
QMF isolation range	None	m/z 6300 – 9300	m/z 6300 – 9300
HCD collision voltage (V)	None	None	100
Max. Injection time (ms)	750	750	750
AGC target (charges)	$5x10^6$	$5x10^6$	$5x10^6$
Orbitrap scan range	m/z 6300 – 9300	m/z 6300 – 9300	m/z 6300 – 9300
Orbitrap resolution (fwhm @ m/z 200)	7500	7500	7500
Microscans	4	4	4

Table S2: Experimental settings for Q-HCD voltage optimisation experiments.

Setting	Mouse brain	Rat kidney
Nano-DESI flow rate (µL/min)	0.65	0.65
Nano-DESI probe velocity (µm/s)	3	3
Nano-DESI spray voltage (V)	1350	1450
In-source CID (V)	200	185
Source CID compensation factor	0.07	0.07
QMF isolation range	m/z 6300 – 9300	m/z 6000 – 9000
HCD collision voltage (V)	0 – 150	0 – 150
HCD voltage step (V)	10	10
Orbitrap resolution (fwhm @ m/z 200)	7500	7500
Orbitrap scan range	m/z 1000 – 16000	m/z 1000 – 16000
Max. Injection time (ms)	750	750
AGC target (charges)	$5x10^6$	$5x10^6$
Microscans	4	4

Table S3: Instrument method details for nano-DESI MSI

Setting	Mouse Cerebellum	Rat Kidney
Nano-DESI flow rate (µL/min)	0.65	0.65
Nano-DESI probe velocity (µm/s)	4	7
Nano-DESI spray voltage (V)	1300	1500
In-source CID (V)	190	185
Source CID compensation factor	0.070	0.070
Isolation mode	Quadrupole	Quadrupole
Isolation range	m/z 6300 – 9300	m/z 6000 – 9000
Activation type	HCD	HCD
HCD collision voltage (V)	100	70
Max. Injection time (ms)	750	1500
AGC target (charges)	$2.5x10^6$	$5x10^6$
Orbitrap scan range	6000 – 9300	6000 – 9000
Orbitrap resolution FWHM (@m/z 200)	7500	7500
Microscans	4	4

Table S4: MetaUniDec settings for deconvolution of the mouse cerebellum nano-DESI MSI.

Parameter	Value
**Data processing**	
m/z range	6000 – 9026
Background subtraction	0
Bin Every	0
Data reduction (%)	0
Intensity threshold	0
Normalize data	Unchecked
**UniDec Parameters**	
Charge range	14 – 35
Mass range (Da)	120,000 – 250,000
Sample mass every (Da)	1
**Quick controls**	
Smooth charge states distributions	Checked
Use automatic m/z peak width	Unchecked
Smooth nearby points	Some
Suppress artifacts	None
Mass differences (Da)	Unchecked
**Peak selection, extraction, and plotting**	
Picking range (Da)	100
Picking threshold	0.1
Peak normalization	Max
How to extract peaks	Height
Extraction window	0
Extraction threshold	10

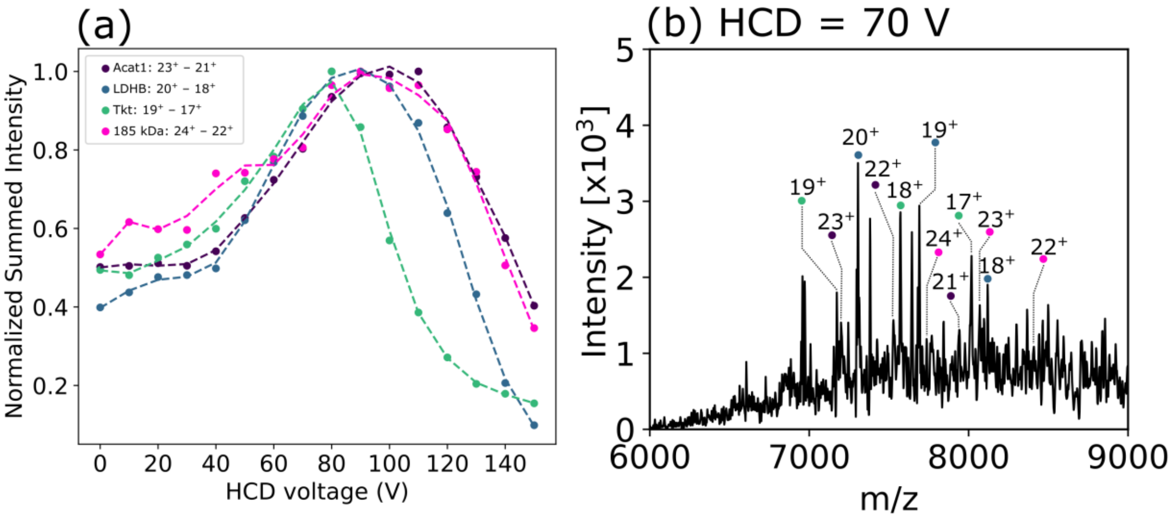

Figure S3: (a) HCD voltage optimisation for protein complexes in rat kidney. Three charge states for each protein complex were monitored and their intensities summed and normalised. Acat1: 23+, 22+, 21+. LDHB: 20+, 19+, 18+. Transketolase: 19+, 18+, 17+. 185 kDa protein: 24+, 23+, 22+. (b) nano-DESI-HCD mass spectrum at HCD = 70 V. 70 V avoided detrimental effects to transketolase signal. Trendlines: Savitzky-Golay filter (window = 5, polynomial = 3). Note that Acat1 is not discussed in the main manuscript, but was identified by nano-DESI nTDMS using information in Figure S13 and Table S15.

Table S5: mouse brain protein complexes identified in this work.

Protein	Uniprot	Calculated MW (Da)	MW from image[a]	Replicate error (Da)[b]	Measured MW (PTCR) (Da)	Calculated MW (monomer) (Da)	Measured MW (monomer, HCD) (Da)
**PKM1 tetramer**	P52480-2	231418	231414	10	231418	57854	57852
**AldoC tetramer**	P05063	157056	157058	30	157087	39264	N/D
**$LDHB_4$ tetramer**	P16125	145934	145926	6	145933	36483	36481
**$LDHA_1B_3$ tetramer**	P16125 + P06151	146165	146212	40	146181	(GS-LDHA) 36715<br>(LDHB) 36483	(GS-LDHA) 36714<br>(LDHB) 36481
**GPI homodimer**	P06745	125357	125357	6	125357	62678	N/D

[a]peak centroid from MetaUniDec image processing. [b]weighted standard deviation for deconvolution across all pixels. N/D: not detected.

Table S6: rat kidney protein complexes identified in this work.

Protein	Uniprot	Ligand/cofactor	Calculated MW (Da)	Measured MW (PTCR) (Da)	Calculated MW (monomer) (Da)	Measured MW (monomer, HCD) (Da)
**$LDHA_4$ tetramer**	P04642	N/A	145447	145430	36362	36361
**$LDHB_4$ tetramer**	P42123	N/A	146094	146092	36524	36527
**$LDHA_1B_3$ tetramer**	P04642 + P42123	N/A	145932	145935	(LDHA) 36362<br>(LDHB) 36524	36362<br>36527
**CGL tetramer**	P18757	pyridoxal phosphate	175400	175584	(apo) 43605<br>(holo) 43850	43620 (apo)<br>43850 (holo)
**Tkt homodimer**	P50137	2x thiamine pyrophosphate, 2x Mg^{2+}	136264	136278	(apo) 67686	N/D
**Acat1 tetramer**	P17764	N/A	165461	165507	41365	41364

N/A: not applicable. N/D: not detected.

Protein identification.

Mouse brain

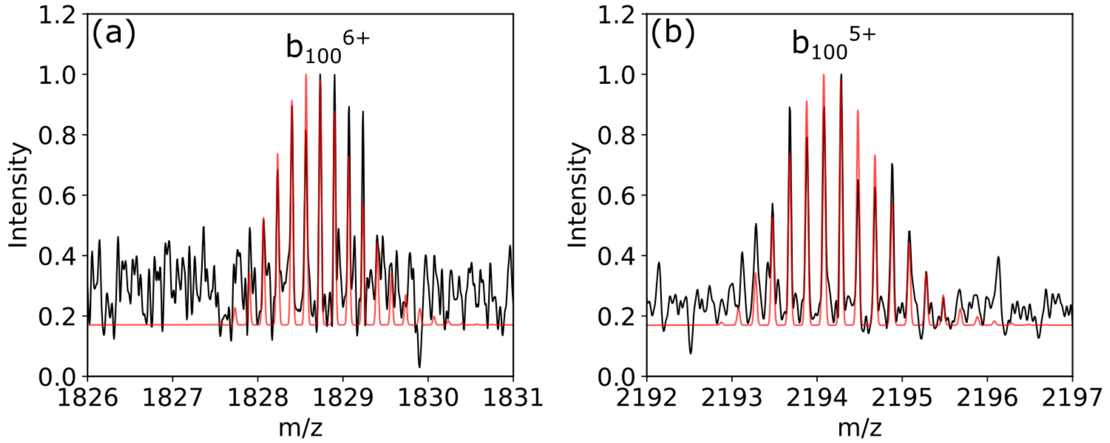

(c)

```
N   P K P H S E A G T A F I Q T Q Q L H A A M A D T F  25
26  L E H M C R L D I D S A P I T A R N T G I I C T I  50
51  G P A S R S V E M L K E M I K S G M N V A R L N F  75
76  S H G T H E Y H A E T I K N V R E A T E S F A S D 100
101 P I L Y R P V A V A L D T K G P E I R T G L I K G 125
126 S G T A E V E L K K G A T L K I T L D N A Y M E K 150
151 C D E N I L W L D Y K N I C K V V E V G S K I Y V 175
176 D D G L I S L Q V K E K G A D F L V T E V E N G G 200
201 S L G S K K G V N L P G A A V D L P A V S E K D I 225
226 Q D L K F G V E Q D V D M V F A S F I R K A A D V 250
251 H E V R K V L G E K G K N I K I I S K I E N H E G 275
276 V R R F D E I L E A S D G I M V A R G D L G I E I 300
301 P A E K V F L A Q K M M I G R C N R A G K P V I C 325
326 A T Q M L E S M I K K P R P T R A E G S D V A N A 350
351 V L D G A D C I M L S G E T A K G D Y P L E A V R 375
376 M Q H L I A R E A E A A M F H R L L F E E L V R A 400
401 S S H S T D L M E A M A M G S V E A S Y K C L A A 425
426 A L I V L T E S G R S A H Q V A R Y R P R A P I I 450
451 A V T R N P Q T A R Q A H L Y R G I F P V L C K D 475
476 A V L N A W A E D V D L R V N L A M D V G K A R G 500
501 F F K K G D V V I V L T G W R P G S G F T N T M R 525
526 V V P V P C
```

Figure S4: Signals for mouse PKM1 sequence ion b_{100} in (a) 6+ charge state and (b) 5+ charge state overlaid with their simulated mass spectra (red trace). (c) The sequence of PKM1. Sequence ion b_{100} occurs at the sole occurrence of "DP" in the sequence, which has the highest propensity for formation during collisional activation.[1]

Table S7: b_{100} sequence ion of PKM1.

Ion	Monoisotopic Mass (Da)	Calculated Mass (Da)	Error (ppm)
**b100**	10959.3453	10959.3396	0.5

```
N   P H S Y P A L S A E Q K K E L S D I A L R I V T P  25
26  G K G I L A A D E S V G S M A K R L S Q I G V E N  50
51  T E E N R R L Y R Q V L F S A D D R V K K C I G G  75
76  V I F F H E T L Y Q K D D N G V P F V R T I Q D K 100
101 G I L V G I K V D K G V V P L A G T D G E T T T Q 125
126 G L D G L L E R C A Q Y K K D G A D F A K W R C V 150
151 L K I S D R T P S A L A I L E N A N V L A R Y A S 175
176 I C Q Q N G I V P I V E P E I L P D G D H D L K R 200
201 C Q Y V T E K V L A A V Y K A L S D H H V Y L E G 225
226 T L L K P N M V T P G H A C P I K Y S P E E I A M 250
251 A T V T A L R R T V P P A V P G V T F L S G G Q S 275
276 E E E A S L N L N A I N R C P L P R P W A L T F S 300
301 Y G R A L Q A S A L N A W R G Q R D N A G A A T E 325
326 E F I K R A E M N G L A A Q G R Y E G S G D G G A 350
351 A A Q S L Y I A N H A Y C
```

Figure S5: Sequence ions for mouse AldoC detected by nano-DESI-HCD MS^2 of m/z 7481±2.5. HCD voltage range 135 – 147 V.

Table S8: Detected sequence ions for mouse AldoC.

Ion	Monoisotopic Mass (Da)	Calculated Mass (Da)	Error (ppm)
**y15**	1505.7236	1505.7263	-1.8
**b66**	7233.7959	7233.7995	-0.5
**b87**	9711.0933	9711.0885	0.5
**b109**	12075.4173	12075.4049	1.0
**b113**	12458.6265	12458.6582	-2.5
**y123**	13058.5476	13058.5434	0.3
**y124**	13161.5365	13161.5527	-1.2
**y128**	13523.7216	13523.7232	-0.1
**b128**	13915.2582	13915.3263	-4.9
**y133**	14065.9855	14065.9750	0.7
**y179**	19262.7144	19262.7072	0.4
**b229**	25173.1898	25173.2195	-1.2
**y234**	25323.8736	25323.8676	0.2

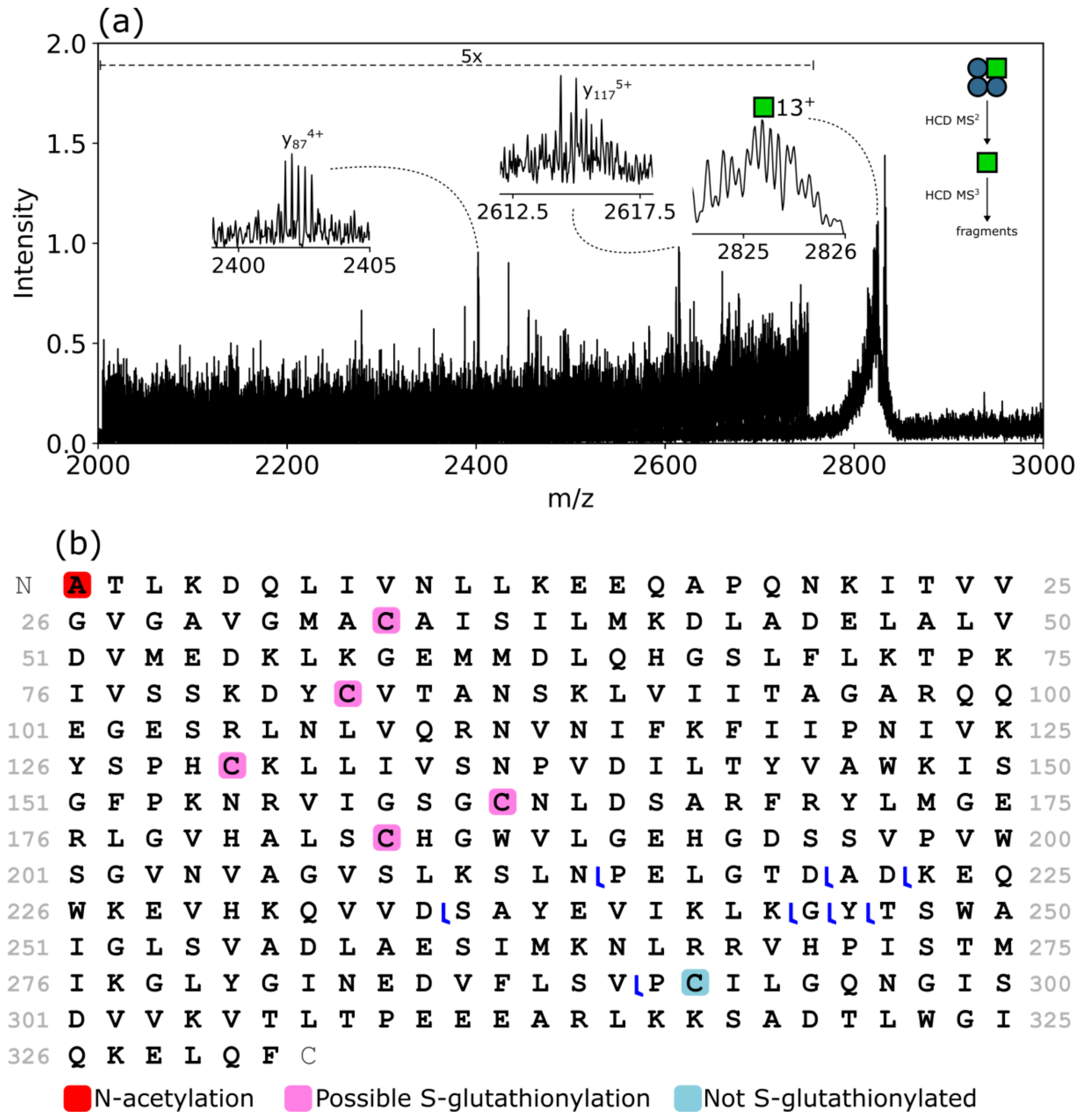

Figure S6: (a) nano-DESI-HCD MS3 of mouse LDHA subunit, m/z 7250±1000 → HCD MS3 m/z 2825^{13+}±25. Note that low abundance of this product ion made MSn challenging. (b) Map of detected sequence ions. All sequence ions were y-ions. The LDHA subunit differed in MW by ~304 Da compared to the calculated MW. Five cysteine residues (pink) are marked as possible sites for glutathionylation (= 305 Da). y-ions suggest C292 (pale blue), towards the C-terminus, was unmodified.

Table S9: detected sequence ions for LDHA

Ion	Monoisotopic Mass (Da)	Calculated Mass (Da)	Error (ppm)
**y41**	4553.4130	4553.4203	-1.6
**y86**	9541.9920	9542.0498	-6.1
**y87**	9599.0868	9599.0712	-1.6
**y88**	9727.1712	9727.1244	-4.8
**y96**	10630.6387	10630.6728	-3.2
**y109**	12264.5390	12264.5305	-0.7
**y111**	12451.6431	12451.5944	-3.9
**y117**	13062.8541	13062.8699	-1.2

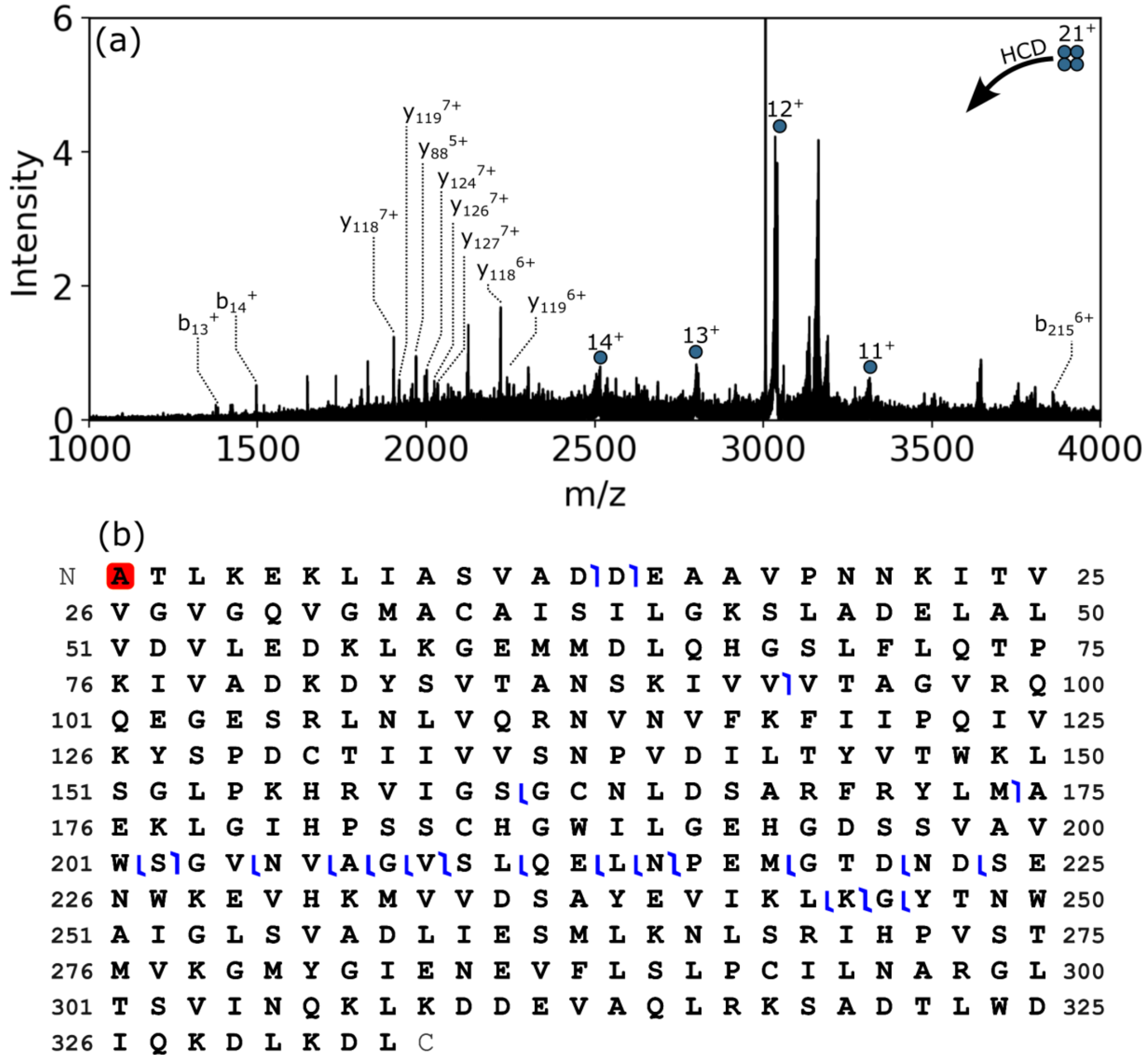

Figure S7: (a) nano-DESI-HCD MS^2 spectrum of the mouse LDHB$_4$ complex (m/z 7297^{20+} ±2.5, 135 V). (b) sequence ion map for mouse LDHB. The N-terminus is acetylated.

Table S10: sequence ions for mouse $LDHB_4$.

Ion	Monoisotopic Mass (Da)	Calculated Mass (Da)	Error (ppm)
**b13**	1381.7780	1381.7817	-2.7
**b14**	1496.8046	1496.8086	-2.7
**y87**	9783.1108	9783.1547	-4.5
**y88**	9841.1416	9841.1762	-3.5
**y89**	9968.2175	9968.2711	-5.4
**b93**	9783.1108	9783.2143	-10.6
**y110**	12451.4692	12451.5386	-5.6
**y112**	12682.4882	12682.6085	-9.5
**y115**	12955.6725	12955.7046	-2.5
**y118**	13313.8412	13313.8404	0.1
**y119**	13426.8288	13426.8833	-4.1
**y120**	13538.9861	13538.9674	1.4
**y122**	13799.0116	13799.0686	-4.1
**y124**	13997.1263	13997.1847	-4.2
**y125**	14098.2219	14098.2531	-2.2
**y126**	14153.2560	14153.2745	-1.3
**y127**	14224.2555	14224.3116	-3.9
**y129**	14439.3538	14439.4230	-4.8
**y132**	14679.4880	14679.5449	-3.9
**y172**	19062.3146	19062.6227	-16.2
**b174**	18924.3402	18924.1129	12.0
**b202**	21865.7835	21865.5105	12.5
**b209**	22460.9805	22460.8387	6.3
**b215**	23144.1549	23144.1830	-1.2
**b245**	26617.8311	26617.8472	-0.6

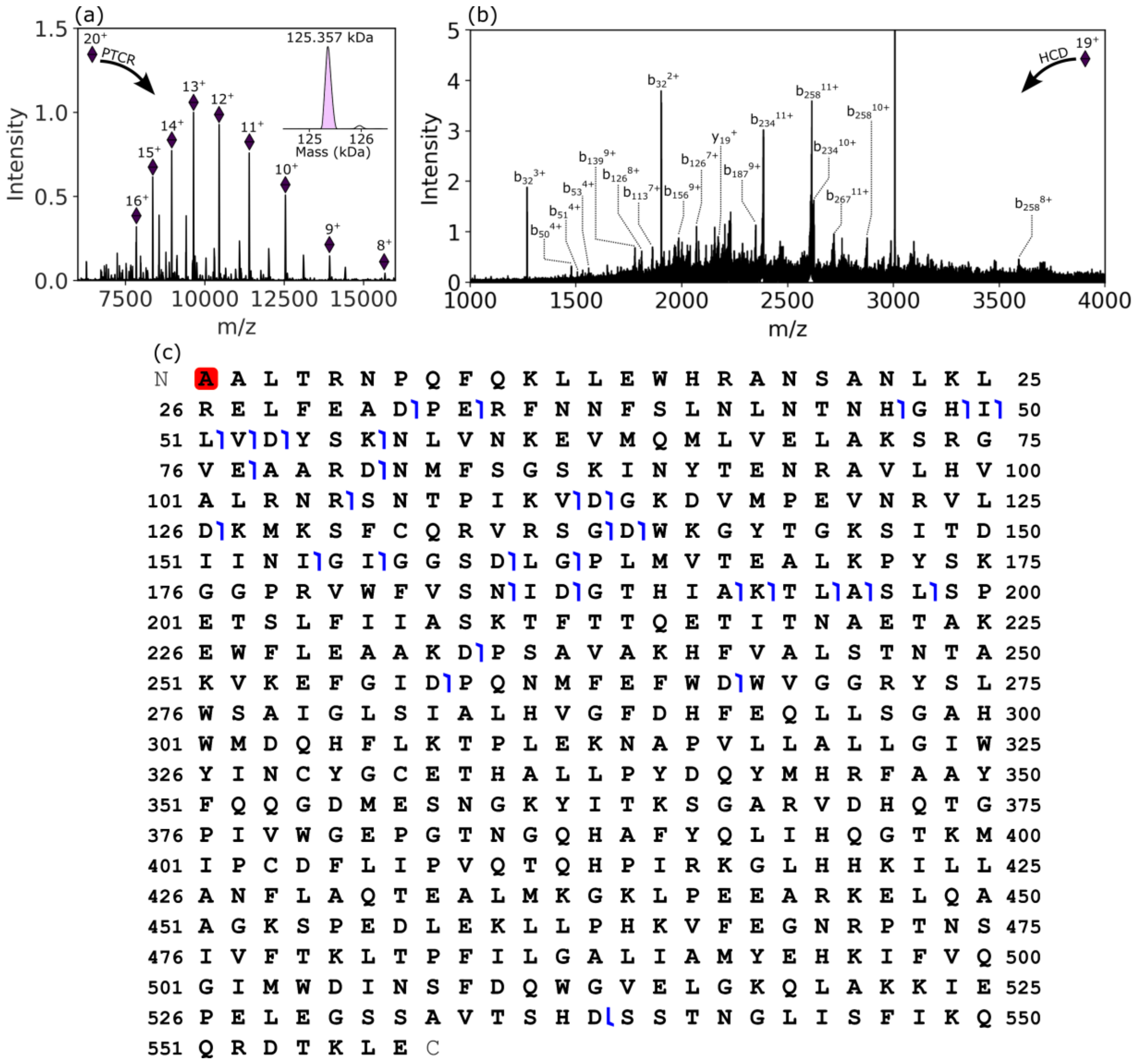

Figure S8: nano-DESI MS² analysis of mouse GPI. (a) PTCR MS² of m/z 6269²⁰⁺ (40 ms reaction time) to determine intact molecular weight. (b) HCD MS² of m/z 6598¹⁹⁺ to obtain sequence information via fragment ions. (c) sequence ion map for mouse GPI. The red highlight indicates N-terminal acetylation.

Table S11: Sequence ions for mouse GPI.

Ion	Monoisotopic Mass (Da)	Calculated Mass (Da)	Error (ppm)
**b32**	3802.9945	3803.0213	-7.0
**b34**	4028.0798	4028.1167	-9.2
**b47**	5600.8454	5600.8760	-5.5
**b49**	5794.9166	5794.9564	-6.9
**b50**	5907.9822	5908.0404	-9.8
**b51**	6021.1019	6021.1245	-3.8
**b52**	6121.1476	6121.1929	-7.4
**b53**	6234.1855	6234.2198	-5.5
**b56**	6612.3677	6612.4102	-6.4
**b77**	8982.6148	8982.6708	-6.2
**b81**	9394.7891	9394.8731	-8.9
**b105**	12164.2448	12164.3132	-5.6
**b112**	12906.5870	12906.7361	-11.6
**b113**	13019.6744	13019.7630	-6.8
**b126**	14471.3986	14471.5025	-7.2
**b138**	15879.0418	15879.2252	-11.6
**b139**	15994.1273	15994.2522	-7.8
**b154**	17685.0686	17685.1611	-5.2
**b156**	17855.1482	17855.2667	-6.6
**b160**	18172.2763	18172.3685	-5.1
**b162**	18341.3525	18341.4741	-6.6
**b185**	20899.8364	20899.8256	0.5
**b187**	21126.8484	21126.9366	-4.2
**b192**	21605.1311	21605.1858	-2.5
**b193**	21735.2234	21735.2807	-2.6
**b195**	21948.3232	21948.4125	-4.1
**b196**	22018.3985	22018.4496	-2.3
**b198**	22219.4007	22219.5657	-7.4
**b234**	26219.4739	26219.5650	-3.5
**b258**	28730.9080	28730.9135	-0.2
**b267**	29925.3134	29925.3939	-2.7

Rat kidney

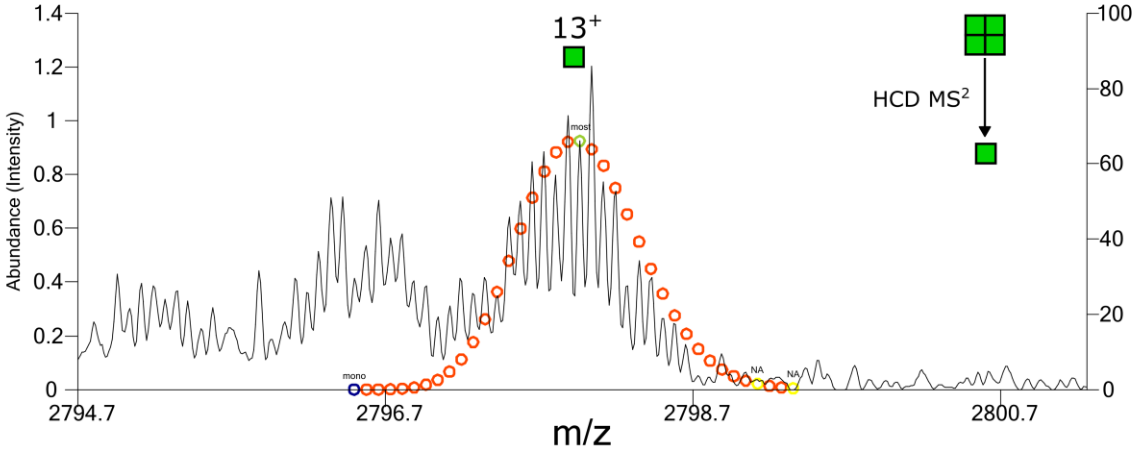

Figure S9: Isotopic distribution of LDHA 13+ subunit ejected from LDHA$_4$, HCD m/z 6928^{21+}. Measured MW = 36360 Da, calculated MW = 36362 Da.

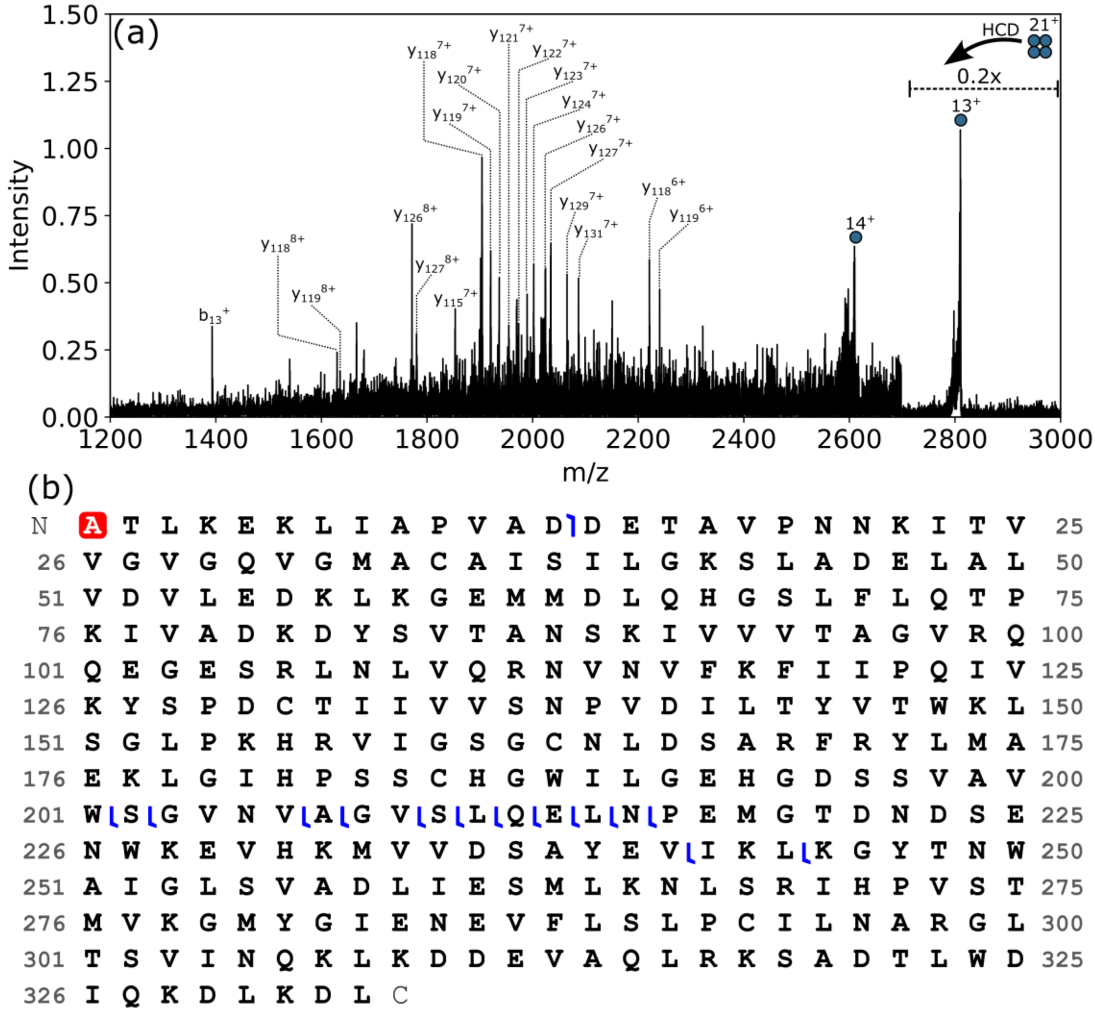

Figure S10: (a) nano-DESI-HCD MS^2 spectrum of m/z 6960^{21+}±2.5 showing LDHB subunits and sequence ions. (b) sequence ion coverage of rat LDHB. The N-terminus is acetylated.

Table S12: detected sequence ions for rat LDHB.

Ion	Monoisotopic Mass (Da)	Calculated Mass (Da)	Error (ppm)
**b13**	1391.7982	1391.8024	-3.0
**y89**	9969.2660	9969.2711	-0.5
**y92**	10323.5266	10323.5342	-0.7
**y118**	13313.7886	13313.8404	-3.9
**y119**	13427.8353	13427.8833	-3.6
**y120**	13539.8749	13539.9674	-6.8
**y121**	13668.9631	13669.0100	-3.4
**y122**	13797.9710	13798.0686	-7.1
**y123**	13912.0700	13912.1526	-5.9
**y124**	13998.1513	13998.1847	-2.4
**y126**	14154.2516	14154.2745	-1.6
**y127**	14225.2333	14225.3116	-5.5
**y131**	14593.6196	14593.5128	7.3
**y132**	14682.5584	14682.5449	0.9

```
N   M E G Y H K P D Q Q K L Q A L K D T A N R L R I S  25
26  S I Q A T T A A G S G H P T S C C S A A E I M A V  50
51  L F F H T M R Y K A L D P R N P H N D R F V L S K  75
76  G H A A P I L Y A V W A E A G F L P E A E L L N L  100
101 R K I S S D L D G H P V P K Q A F T D V A T G S L  125
126 G Q G L G A A C G M A Y T G K Y F D K A S Y R V Y  150
151 C M L G D G E V S E G S V W E A M A F A G I Y K L  175
176 D N L V A I F D I N R L G Q S D P A P L Q H Q V D  200
201 V Y Q K R C E A F G W H A I I V D G H S V E E L C  225
226 K A F G Q A K H Q P T A I I A K T F K G R G I T G  250
251 I E D K E A W H G K P L P K N M A E Q I I Q E I Y  275
276 S Q V Q S K K K I L A T P P Q E D A P S V D I A N  300
301 I R M P T P P N Y K V G D K I A T R K A Y G L A L  325
326 A K L G H A S D R I I A L D G D T K N S T F S E L  350
351 F K K E H P D R F I E C Y I A E Q N M V S I A V G  375
376 C A T R D R T V P F C S T F A A F F T R A F D Q I  400
401 R M A A I S E S N I N L C G S H C G V S I G E D G  425
426 P S Q M A L E D L A M F R S V P M S T V F Y P S D  450
451 G V A T E K A V E L A A N T K G I C F I R T S R P  475
476 E N A I I Y S N N E D F Q V G Q A K V V L K S K D  500
501 D Q V T V I G A G V T L H E A L A A A E M L K K E  525
526 K I G V R V L D P F T I K P L D K K L I L D C A R  550
551 A T K G R I L T V E D H Y Y E G G I G E A V S A V  575
576 V V G E P G V T V T R L A V S Q V P R S G K P A E  600
601 L L K M F G I D K D A I V Q A V K G L V T K G C
```

Figure S11: sequence ion coverage for the sequence of rat transketolase. The N-terminus is acetylated.

Table S13: detected sequence ions for rat transketolase.

Ion	Monoisotopic Mass (Da)	Calculated Mass (Da)	Error (ppm)
**y62**	6390.4502	6390.4776	-4.3
**b62**	6831.3409	6831.3604	-2.8
**y76**	7905.2693	7905.2811	-1.5
**y90**	9526.2551	9526.2617	-0.7
**y108**	11493.2688	11493.4062	-12.0
**y109**	11561.3267	11561.4433	-10.1
**y111**	11827.5235	11827.5448	-1.8
**y113**	12041.6467	12041.6765	-2.5
**y115**	12197.6722	12197.7664	-7.7
**y116**	12271.8395	12271.8035	2.9
**y117**	12325.8198	12325.8250	-0.4
**y118**	12438.8772	12438.9090	-2.6
**y120**	12639.0499	12639.0251	2.0
**y123**	12982.1505	12982.1791	-2.2
**y173**	18385.9483	18386.0039	-3.0
**y199**	21243.2576	21243.3164	-2.8

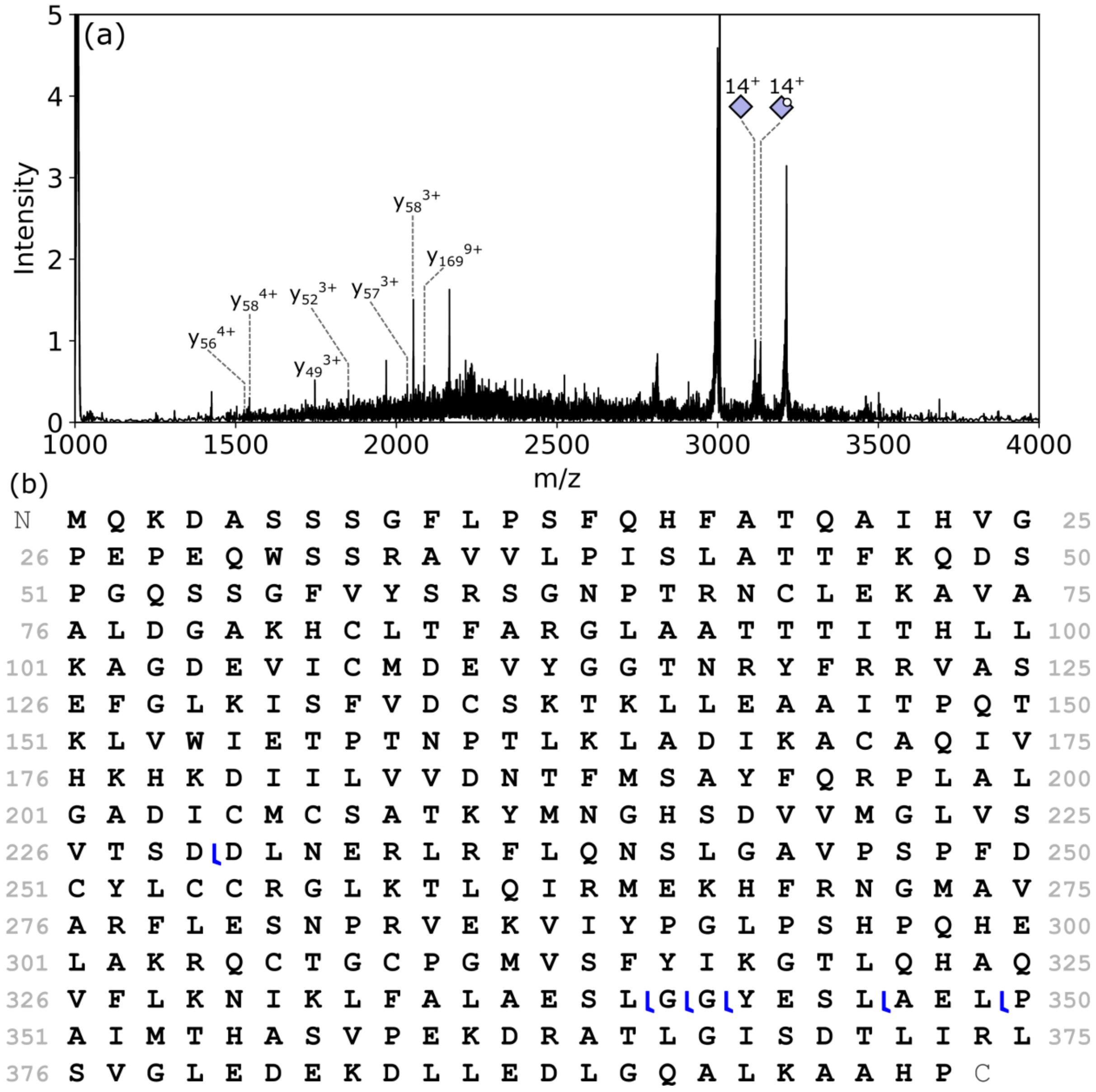

Figure S12: (a) nano-DESI-HCD MS^2 spectrum of rat CGL (m/z 7636^{23+}±10). (b) Sequence ion coverage of rat CGL.

Table S14: detected sequence ions for rat CGL.

Ion	Monoisotopic Mass (Da)	Calculated Mass (Da)	Error (ppm)
**y49**	5233.7524	5233.7655	-2.5
**y52**	5546.9186	5546.9293	-1.9
**y56**	6039.1660	6039.1512	2.5
**y57**	6096.1758	6096.1728	0.5
**y58**	6153.1763	6153.1942	-2.9
**y169**	18760.6776	18760.7374	-3.2

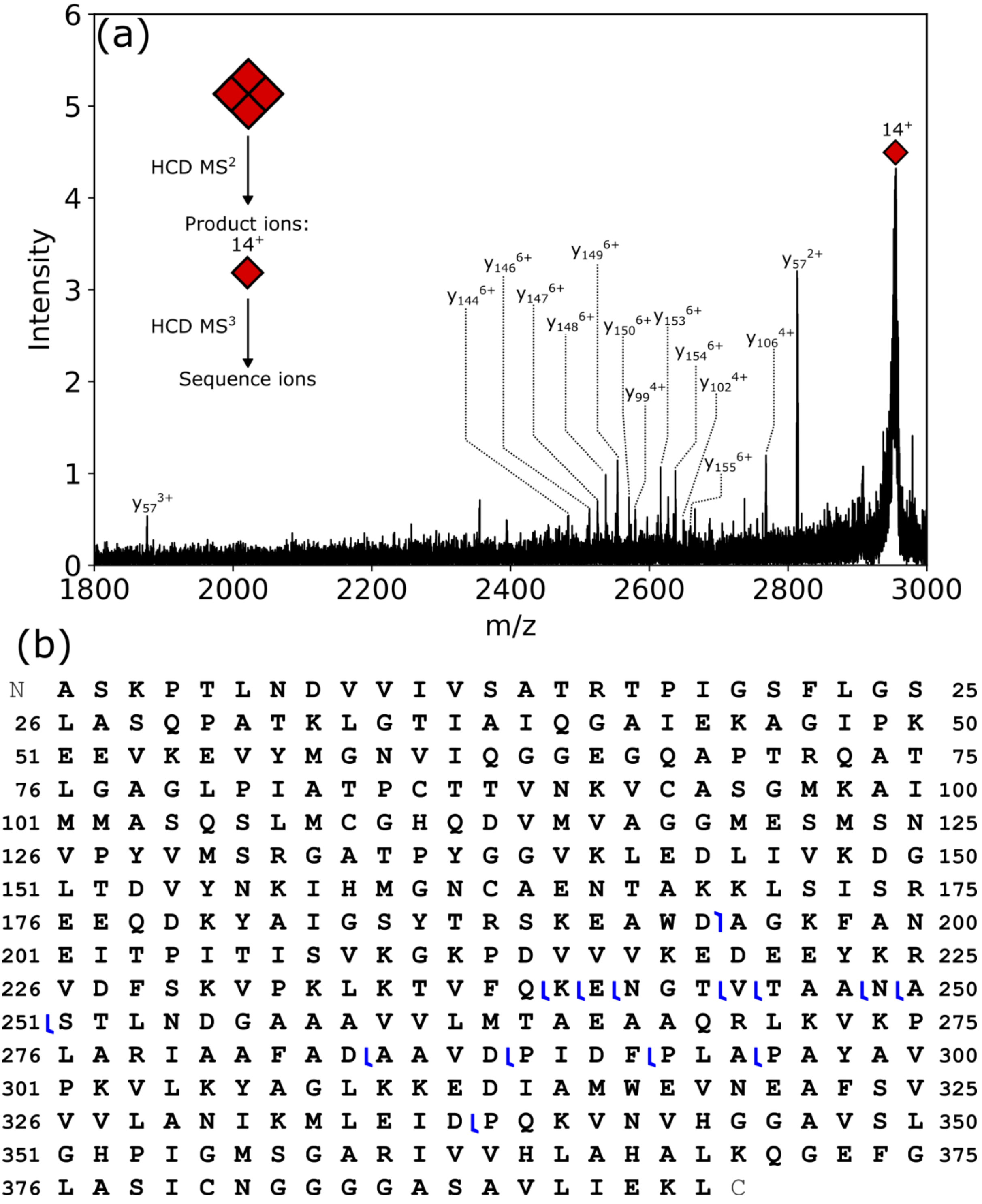

Figure S13: HCD MS^3 spectrum and sequence ions for Acat1 (a) HCD m/z 6000±1000 (50 V)→ HCD m/z 2955^{14+}±25 (106 V). (b) MS^3 product ions assigned to the Acat1 amino acid sequence.

Table S15: detected sequence ions for rat Acat1.

Ion	Monoisotopic Mass (Da)	Calculated Mass (Da)	Error (ppm)
**b194**	20341.1315	20341.2077	-3.7
**y57**	5622.0056	5622.0190	-2.4
**y57**	5622.0061	5622.0190	-2.3
**y99**	10308.5362	10308.5398	-0.4
**y102**	10589.6595	10589.7138	-5.1
**y106**	11061.9682	11061.9459	2.0
**y153**	15681.2416	15681.3952	-9.8
**y153**	15681.3864	15681.3952	-0.6
**y154**	15810.3773	15810.4378	-3.8
**y155**	15938.4427	15938.5327	-5.6
**b194**	20341.1315	20341.2077	-3.7
**y110**	11417.1039	11417.1155	-1.0
**y110**	11417.1619	11417.1155	4.1
**y144**	14880.9313	14881.0128	-5.5
**y145**	14951.9940	14952.0499	-3.7
**y146**	15066.0648	15066.0928	-1.9
**y147**	2524.0330	2524.0290	1.6
**y148**	2535.8730	2535.8685	1.8
**y149**	15309.1840	15309.2147	-2.0
**y150**	15408.2330	15408.2831	-3.3
**y155**	15937.5646	15937.5327	2.0

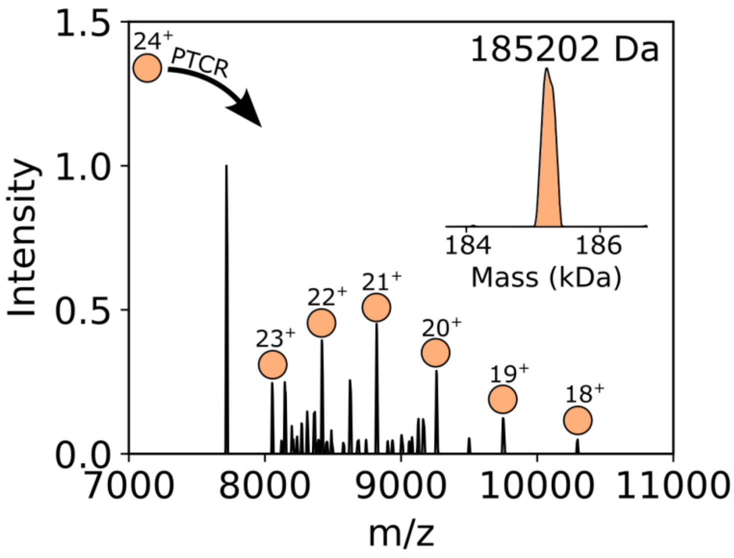

Figure S14: nano-DESI-PTCR MS^2 spectrum of m/z 7718^{24+}±7.5 (reaction time = 1.5 ms) and the deconvoluted mass spectrum inset.

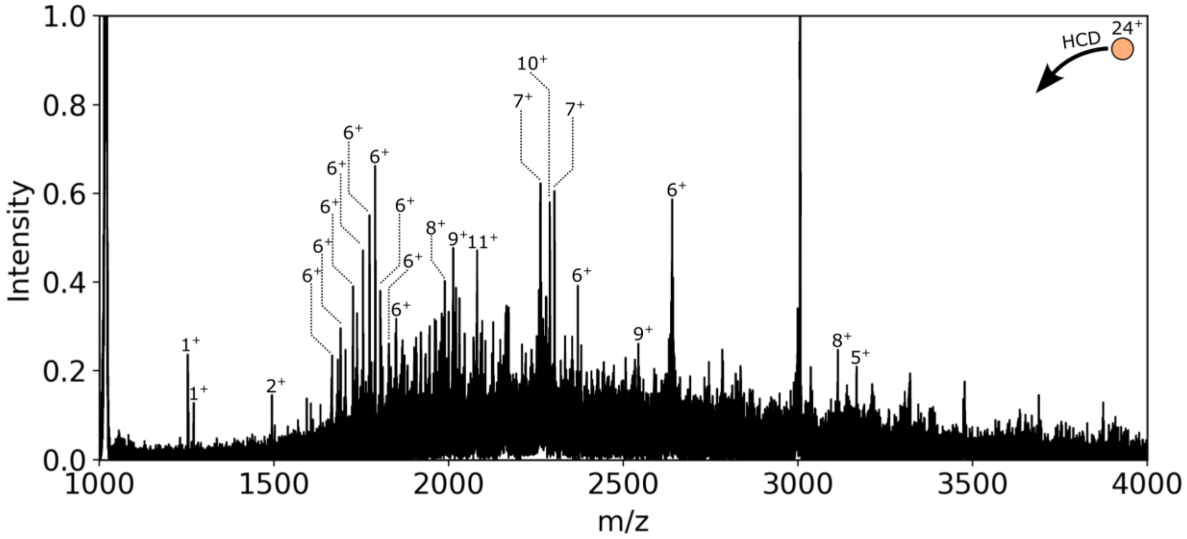

Figure S15: nano-DESI-HCD MS^2 spectrum of m/z 7718^{24+}±5 (HCD voltage = 140 V).

References

(1) Ives, A. N.; Su, T. J. F.; Durbin, K. R.; Early, B. P.; Seckler, H. D.; Fellers, R. T.; LeDuc, R. D.; Schachner, L. F.; Patrie, S. M.; Kelleher, N. L. Using 10,000 Fragment Ions to Inform Scoring in Native Top-down Proteomics *J Am Soc Mass Spectrom* **2020**, *31*, 1398-1409, 10.1021/jasms.0c00026
